# Platelets from older adults exhibit differences in mitochondrial function associated with impaired glucose metabolism

**DOI:** 10.1042/CS20242841

**Published:** 2026-01-14

**Authors:** Gargi Mahapatra, Zhengrong Gao, James R. Bateman, Samuel Neal Lockhart, Jaclyn Bergstrom, Jemima Elizabeth Piloso, Suzanne Craft, Anthony J. A. Molina

**Affiliations:** 1 Division of Geriatrics, Gerontology, and Palliative Care; Department of Medicine, University of California San Diego School of Medicine, La Jolla, CA, U.S.A.; 2 Section on Gerontology and Geriatrics, Sticht Center for Healthy Aging and Alzheimer’s Prevention, Department of Internal Medicine, Wake Forest Baptist Medical Center, Winston-Salem, NC, U.S.A.; 3 Department of Neurology, Wake Forest Baptist Medical Center, Winston-Salem, NC, U.S.A.

**Keywords:** diabetes, impaired glucose tolerance, insulin resistance, mitochondrial bioenergetics, normoglycemia, platelets

## Abstract

Impaired glucose tolerance (IGT) and insulin resistance (IR), including prediabetes and diabetes, increase risk of developing age-related disorders, such as cardiovascular disorders, kidney disorders, and Alzheimer’s disease. We analyzed mitochondrial bioenergetics of platelets collected from 208 adults, 55 years and older, with IGT and IR and without normoglycemic (NG). Platelets from IGT participants exhibited unique mitochondrial bioenergetic profiles exemplified by higher mitochondrial respiration compared with NG. IGT platelets exhibited higher glucose-dependent maximal respiration (Max) and spare respiratory capacities (SRCs) and higher fatty acid oxidation (FAO)-dependent maximal coupled (MaxOXPHOS) and uncoupled (maximal electron transport system) respiration compared with NG. Correlating mitochondrial bioenergetics from all 208 participants with measures of glucose tolerance (oral glucose tolerance test values measured 120 min after glucose administration, and oral glucose tolerance test area under the curve), and historical glucose measures [hemoglobin A1 (HbA1c)] revealed significant positive associations. Most associations were unaltered with age, sex, and body mass index adjustments. Examining NG and IGT participants separately, we found platelet respiration and HbA1c exhibited positive association in NG participants. Significant positive associations emerged between platelet SRC, FAO, FAO+CI (oxygen flux due to FAO + complex I activities), and HbA1c. No significant associations were observed in the IGT group. Given the utilization of blood-based mitochondrial bioenergetic profiling strategies in clinical research, this work provides new insights into the clinical features of IR that can affect platelet mitochondrial bioenergetics.

## Introduction

The prevalence of type-2 diabetes mellitus (T2DM) is increasing with the aging of the population worldwide [1]. Age is a major driver of diabetes-related disorders, and an estimated 33% of older adults ≥ 65 years in the USA live with diabetes or prediabetes [2]. Adults 65 years and older with diabetes are at increased risk for geriatric disabilities, including functional disabilities, cognitive impairment, frailty and falls, urinary incontinence, depression, and morbidities such as microvascular and cardiovascular diseases [3]. T2DM is a systemic inflammatory disease that is exemplified by insulin resistance (IR) and lower metabolic response to insulin [4]. T2DM and hyperglycemia have been shown to cause increased production of reactive oxygen species (ROS), and increased ROS due to hyperglycemia causes oxidative stress, altering cellular homeostasis and leading to tissue damage [5]. Age is the main risk factor for diseases like cancer, neurodegeneration, and heart disease, and these age-associated disorders are exacerbated with T2DM. Older adults are likely to exhibit cumulative impacts related to aging, as well as living with T2DM or prediabetes. Hyperglycemia affects cellular organelles such as mitochondria, endoplasmic reticulum, and lysosomes [6]. A growing body of evidence suggests that mitochondrial dysfunction is the major mediator of pathogenesis due to T2DM and IR [7]. Mitochondria play a key role in maintaining cellular metabolism and homeostasis by providing cellular energy through adenosine triphosphate (ATP) generation using oxidative phosphorylation (OXPHOS) and are the major source of ROS generation. Mitochondria are also essential for glucose-mediated insulin production and secretion by the pancreatic β-cells [8]. Aging and type 2 diabetes have been individually shown to affect mitochondrial functions. Older adults are more susceptible to systemic mitochondrial functional differences. Consequences of mitochondrial bioenergetic decline due to aging itself and the greater burden of comorbidities are worse in older adults. Therefore, the older adult population with T2DM and prediabetes is an interesting population to study mitochondrial functional alterations as compared with the older adults without T2DM or prediabetes, which is why they are the focus of our study.

Metabolic disorders such as IR and T2DM have been shown to affect mitochondrial function in humans [6]. T2DM-related alterations in mitochondrial function have been observed in different tissues and cells, including adipose tissue, liver, skeletal muscle, and cardiac muscle, as well as peripheral circulatory cells such as platelets [4]. In the resting state, platelet mitochondria are highly active, making platelets highly metabolically active cells in circulation [9]. Mitochondria play a central role in platelet activation and function under homeostatic conditions [9]. Studies of healthy platelets have suggested that under physiological conditions, the global metabolome of platelets is significantly affected due to mitochondrial bioenergetic changes [10]. Mitochondrial dysfunction due to diabetes and hyperglycemia may have pathological effects on platelets, altering their function. However, little is known about platelet mitochondrial bioenergetics among older adults with and without hyperglycemia in the context of IR and T2DM.

In this study, we tested the hypothesis that mitochondrial bioenergetics of platelets differ between normoglycemic (NG) and hyperglycemic older adults. We also examined whether the overall mitochondrial bioenergetic capacity of platelets is associated with the hallmarks of T2DM and if these associations may differ between NG and hyperglycemic older adults. We measured platelet mitochondrial bioenergetic capacities in NG individuals and individuals with impaired glucose tolerance (IGT), including individuals with prediabetes or diabetes. Two complementary mitochondrial bioenergetic profiling approaches were utilized. High-throughput respirometry was performed with intact platelets using glucose as the primary fuel source, and high-resolution respirometry was performed with permeabilized platelets using fatty acid and glucose-metabolism derived substrates. Overall, this study provides evidence that metabolic disorders such as IGT affect platelet mitochondrial bioenergetics.

## Methods

### Participants

A total of 208 cognitively unimpaired participants, consisting of 112 NG and 96 with IGT, 55 years and older, were included from the Wake Forest Alzheimer’s Disease Research Center’s Clinical Core Study. Among 96 IGT participants, 69 were individuals with prediabetes and 27 with diabetes. The Institutional Review Board at Wake Forest Baptist Health approved all procedures performed. Written informed consent was obtained from all participants and their authorized representatives. Staff and key investigators were blinded to the glycemic group of the samples during mitochondrial bioenergetic assessments.

### Determination of glucose tolerance

All participants had fasted for 12 h prior to glucose testing. Glycemic status was determined using an oral glucose tolerance test (OGTT), hemoglobin A1c (HbA1c), or a self-reported history of diabetes requiring medication. For OGTT determination, glucose is measured in blood samples taken prior to administration of a standard glucose load of 75 g, as well as 15, 30, and 120 min after administration. HbA1c was measured through standard whole blood collection. The laboratory values closest in time to the blood sample collection for mitochondrial bioenergetics assessments were used. IGT refers to samples that met criteria for either diabetes or prediabetes on HbA1c or OGTT values measured 120 min after glucose administration (OGTT_120). Prediabetes was defined as HbA1c between the values of 5.8% and 6.4% or OGTT_120 between 141 mg/dl and 199 mg/dl. Diabetes was defined as HbA1c of 6.5% or higher, or an OGTT_120 of 200 mg/dl or higher [11]. IGT was defined as meeting criteria for either diabetes or prediabetes. OGTT_120 data from 174 individuals (comprising 104 NG+70 IGT participants), OGTT area under the curve (OGTT_AUC) data from 168 individuals (comprising 103 NG+65 IGT participants), and HbA1c data from 139 individuals (comprising 66 NG+73 IGT participants) were utilized in this study.

### Mitochondrial bioenergetics

#### Platelet isolation

Venous blood was collected from overnight fasted participants in acid citrate dextrose tubes (Vacutainer; Becton Dickinson, Franklin Lakes, NJ), and samples were immediately processed for platelet isolation [12]. Whole blood was centrifuged at 500 × g for 15 min without brake, plasma removed from the tubes after spin, and plasma was centrifuged at 1500 × g for 10 min without brake to isolate platelets. Isolated platelets were then resuspended in 4 ml of PBS containing 1 µM of prostaglandin E1 (PGE1; Cayman Chemical, Ann Arbor, MI) for washing and centrifuged at 1500 × g for 7 min without brake. Washed platelets were resuspended in Seahorse Biosciences extracellular flux (XF) assay buffer containing 1 mM sodium pyruvate, 1 mM glutaMAX (Gibco, Grand Island, NY), 11  mM D-glucose, and 1 µM PGE1 (pH 7.4). Isolated platelets were counted by Coulter AC.Tdiff2 (Beckman Coulter, U.S.A.).

#### Platelet respirometry

High-throughput respirometry was performed with Seahorse XFe24 analyzer (Agilent Technologies, Inc., Santa Clara, CA) to assess intact platelet respiration. Twenty-five million platelets were plated in each well in quadruplicate. Basal oxygen consumption rate (OCR) was followed by sequential injections of oligomycin (0.75 μM), carbonyl cyanide-4-(trifluoromethoxy) phenylhydrazone (FCCP, 1 μM), and antimycin A + rotenone (A/R, 1 μM each). Outcomes were calculated after subtracting non-mitochondrial OCR from all measured parameters. Leak respiration was obtained by subtracting A/R from oligomycin, maximal OCR was obtained by FCCP addition (Max), spare respiratory capacity (SRC) was obtained by subtracting basal respiration from Max, and ATP-linked respiration was obtained by subtracting oligo from basal respiration. Respiration was measured in pmol·min^−1^·25 million^−1^ platelets.

High-resolution respirometry was performed using Oroboros O2K oxygraph (Oroboros, Innsbruck, Austria) to assess permeabilized platelet respiration that is mediated by fatty acid oxidation (FAO) and FAO-dependent metabolism. Mitochondrial OXPHOS was assessed in 200 million platelets per chamber of Oroboros oxygraph following the substrate-uncoupler-inhibitor titration protocol using mitochondrial respiration medium (0.5 mM EGTA, 3 mM MgCl2, 60 mM lactobionic acid, 20 mM taurine, 10 mM KH2PO4, 20 mM HEPES, 110 mM D-Sucrose, 1 g/l fatty acid free BSA, pH 7.1, Supplementary Figure S1 ). Routine endogenous respiration of the cells was assessed, followed by permeabilization of cells using 0.04 mg/ml digitonin and addition of 1 mM ADP and 0.6 mM Mg. Then substrates specific for each electron transport chain (ETC) complex were added sequentially. 0.1 mM malate addition stimulated FAO pathway, 0.5 mM octanoylcarnitine evaluated FAO capacity, 0.01 mM cytochrome c assessed outer mitochondrial membrane integrity, and 2 mM malate kinetically saturated FAO pathway. Then, 5 mM pyruvate and 10 mM glutamate assessed mitochondrial complex I-specific respiration (FAO+CI), 10 mM succinate assessed complex II-specific respiration (FAO+CI+CII), and 10 mM glycerophosphate assessed maximal coupled respiration (FAO+CI+CII+CIII or MaxOXPHOS). ETC was uncoupled by titrating 0.5 μM FCCP until maximal electron transport system (MaxETS) capacity was reached (maximal uncoupled respiration). Next, respiration was inhibited with 1 μM rotenone and 2.5 μM antimycin A, resulting in non-mitochondrial or residual oxygen consumption. Then, 2 mM ascorbate and 0.5 mM tetramethyl-phenylenediamine (TMPD) were added, followed by opening O2K chambers for 20 min and closing for 5 min to record ascorbate and TMPD-mediated oxygen consumption. Finally, the addition of 100 mM azide [complex IV (CIV) inhibitor] completed the experiment. Primary outcomes were calculated after subtracting residual oxygen consumption. Respiration was reported as pmol·sec^−1^·100 million^−1^ platelets.

FCCP titration is performed with an increasing number of 0.5 µM FCCP injections so that we can induce uncoupling of mitochondrial membranes gradually, increasing respiration with each additional injection and reaching maximal respiration without a breakdown of mitochondrial membranes. This leads to capturing the true maximal uncoupled respiration (MaxETS). Addition of a single injection of a very high volume of 0.5 µM FCCP may lead to excess FCCP, beyond the necessary volume needed to reach maximal respiration, which may lead to breakdown of the mitochondrial membranes and prevent us from capturing the true maximal respiration. Therefore, a gradual FCCP titration is performed.

Residual oxygen consumption is defined as the oxygen flux recorded after the addition of antimycin A, a mitochondrial Complex III inhibitor that inhibits any electron transfer from Complex III to cytochrome c, thereby inhibiting the transfer of electrons to CIV and inhibiting the entire ETC. Residual oxygen consumption is the amount of oxygen consumed by cellular enzymes that are unrelated to the mitochondrial ETS. As reported in the methods section, primary outcomes were calculated after subtracting the residual oxygen consumption values.

Ascorbate and *N,N,N′,N′*-tetramethyl-*p*-phenylenediamine (TMPD) are added after inhibiting the ETS with antimycin A to specifically assess mitochondrial CIV enzymatic activity. TMPD is used to reduce cytochrome c, thereby acting as an artificial substrate. However, TMPD rapidly undergoes auto-oxidation in the presence of oxygen in the O2K assay buffer; therefore, ascorbate is added before TMPD to maintain TMPD in a reduced state. Essentially, ascorbate is added just before TMPD to serve as an alternate substrate to be oxidized instead of TMPD, preventing immediate oxidation of TMPD and keeping TMPD in a reduced state. Auto-oxidation of ascorbate and respiration of CIV increase respiration dramatically within the O2K chambers, creating a hypoxic environment. The Oroboros O2k chambers are opened for 20 min to oxygenate the assay buffer and prevent cell hypoxia. Closing the chambers leads to an increase in respiration, eventually reaching a peak that begins to linearly decline, which is recorded for 5 min. This resulting O_2_ flux is recorded for 5 min and includes both ascorbate auto-oxidation and CIV enzymatic activity. Then, CIV is inhibited by the addition of sodium azide, and a respiration measurement is recorded for 10 more min, the resulting O_2_ flux recorded after the addition of sodium azide corresponds to the O_2_ flux due to ascorbate auto-oxidation. To examine CIV-specific activity, the O_2_ flux due to ascorbate auto-oxidation (respiration recorded after sodium azide addition) is subtracted from the peak respiration recorded for 5 min after closing of chambers (O_2_ flux measurements after chamber closure, but prior to the addition of sodium azide).

### Statistical analysis

Shapiro–Wilk tests were performed to assess the normal distribution of all variables, and log transformations were performed for parameters with non-normal distribution. Statistical significance was evaluated by an unpaired t-test (GraphPad Prism 8.4.3, GraphPad Software, Inc., San Diego, CA). Data points are presented as scatter plots with mean ± standard deviation (NG and IGT). Spearman correlation coefficients were assessed between mitochondrial bioenergetic variables and clinical parameters, and partial correlations were assessed with adjustments for age, sex, and body mass index (BMI), using SPSS software (SPSS v22; Armonk, NY). Statistical significance is defined as *P*≤0.05 and indicated with asterisk (*). *P*≤0.1 is highlighted to indicate potential associations. Exact *P* values are reported.

## Results

### Participant characteristics

Demographics and glucose tolerance measures for all participants are summarized in Table 1. The range of each parameter (max and min values), averages, and standard deviations is reported. Our analyses included 112 NG participants and 96 IGT participants, including 69 individuals with prediabetes and 27 individuals with diabetes. The average age was not significantly different between NG (67.96 years) and IGT participants (69.22 years) (*P*=0.25). Average BMI was significantly lower in NG participants (27.03 kg/m^2^) than IGT participants (28.61 kg/m^2^) (*P*=0.04). Average OGTT_120 was significantly lower in NG participants (107.07) than IGT participants (171.63) (*P*=1.638E-30); OGTT_AUC was significantly lower in NG (253.04) compared with IGT participants (323.38) (*P*=8.168E-22). HbA1c was assessed to determine historical glucose measures. HbA1c values were significantly lower in NG (5.47) than IGT participants (5.88) (*P*=2.207E-06). The NG (76% females, 24% males) and IGT (79% females, 21% males) groups comprise similar percentages of females compared with males.

**Table 1:** Participant demographics and glucose measures.

Demographics and glucose tolerance measures	Normoglycemics (NG=112)				Impaired glucose tolerance (IGT=96) (69 PD + 27 D)				*P* values
	Max	Min	Average	Std dev	Max	Min	Average	Std dev	
Age (years)	95.00	55.00	67.96	8.23	92.00	56.00	69.22	7.48	0.25
BMI (kg/m^2^)	46.90	18.20	27.03	5.13	48.30	17.40	28.61	5.84	0.04*
OGTT_120	139.00	58.50	107.07	22.69	255.00	100.50	171.63	29.34	1.638E-30*
OGTT_AUC	335.78	161.11	253.04	31.78	421.06	245.06	323.38	39.80	8.168E-22*
HbA1c	6.30	4.90	5.47	0.31	7.90	4.70	5.88	0.62	2.207E-06*
Sex	Female	Male	Female%	Male%	Female	Male	Female%	Male%	
	85	27	76%	24%	76	20	79%	21%	

Abbreviations: BMI, body mass index; OGTT_120, oral glucose tolerance test 120 min after glucose administration; OGTT_AUC, oral glucose tolerance test area under the curve, HbA1c, Hemoglobin A1c. Values are mean ± standard deviation. *P* values ≤ 0.05 are shown in bold with asterisk (*=≤ 0.05).

As shown in Supplementary Table S7, we separated the IGT group into prediabetics (PD) and diabetic (D) groups. The PD group included 69 participants, and the D group included 27 participants. The average age was not significantly different between NG (67.96 years) and PD participants (68.38 years) (*P*=0.85). However, average age was significantly different between NG (67.96 years) and D participants (70.40 years) (*P*=0.02), and average age was significantly different between PD (68.38 years) and D participants (70.40 years) (*P*=0.04). Average BMI was significantly lower in NG participants (27.03 kg/m^2^) than PD participants (28.29 kg/m^2^) (*P*=0.0004), average BMI was significantly lower in NG participants (27.03 kg/m^2^) than D participants (29.07 kg/m^2^) (*P*=0.005), and average BMI was not significantly different between PD (28.29 kg/m^2^) and D participants (29.07 kg/m^2^) (*P*=0.38). Average OGTT_120 was significantly lower in NG participants (107.07) than PD participants (161.88) (*P*=1.39855E-32), average OGTT_120 was significantly lower in NG participants (107.07) than D participants (204.56) (*P*=4.79553E-16), and average OGTT_120 was significantly lower in PD participants (161.88) than D participants (204.56) (*P*=2.71133E-10). Average OGTT_AUC was significantly lower in NG participants (253.04) than PD participants (310.57) (*P*=3.03635E-17), average OGTT_AUC was significantly lower in NG participants (253.04) than D participants (370.05) (*P*=1.77084E-11), and average OGTT_AUC was significantly lower in PD participants (310.57) than D participants (370.05) (*P*=4.21643E-07). Average HbA1c values were significantly lower in NG participants (5.47) than PD participants (5.66) (*P*=0.0001), average HbA1c values were significantly lower in NG participants (5.47) than D participants (6.11) (*P*=0.0004), and average HbA1c values were significantly lower in PD participants (5.66) than D participants (6.11) (*P*=005). The PD (82.6% females, 17.4% males) and D (70.4% females, 29.6% males) groups comprise similar percentages of females compared with males.

To further delineate the differences in demographic parameters and glucose tolerance measures in participants closer to their age groups, we separated the participants from both the NG and IGT groups into three age-specific subgroups ranging from 55 to 64 years, 65 to 74 years, and 75 years and above, and analyzed the differences in each age subgroup (Supplementary Table S2). Average age was significantly different between NG (59.88 years) and IGT participants (64.35 years) (*P*=0.02) in the 55 to 64 years subgroup, while it was not significant in the 65 to 74 years and 75 years and above subgroups. Average BMI was significantly lower in NG participants (25.01 kg/m^2^) than IGT participants (30.39 kg/m^2^) (*P*=0.001) in the 55 to 64 years subgroup, and significantly lower in NG participants (24.12 kg/m^2^) than IGT participants (27.54 kg/m^2^) (*P*=0.02) in the 75 years and above subgroup, while it was not significant in the 65 to 74 years subgroup. Average OGTT_120 and OGTT_AUC values were significantly lower in the NG participants than IGT participants in all three age subgroups. Specifically, average OGTT_120 values in NG participants (97.57, 109.48, and 118.38) were significantly lower than IGT participants (162.59, 175.34, and 175.74) (*P*=2.992E-09, 8.982E-17, and 9.715E-0.7) in the 55 to 64 years, 65 to 74 years, and 75 years and above age-specific subgroups, respectively; average OGTT_AUC values in NG participants (243.11, 254.97, and 265.04) were significantly lower than IGT participants (308.03, 335.05, and 320.53) (*P*=1.382E-07, 2.968E-12, and 5.574E-0.5) in the 55 to 64 years, 65 to 74 years, and 75 years and above age-specific subgroups. HbA1c values in NG participants (5.47, 5.42, and 5.35) were significantly lower than in IGT participants (5.96, 5.95, and 5.73) (*P*=0.002, 3.459E-05, and 0.03) in the 55 to 64 years, 65 to 74 years, and 75 years and above age-specific subgroups, respectively.

### Mitochondrial bioenergetic profiles of platelets differentiated by glucose tolerance

**Figure 1:**
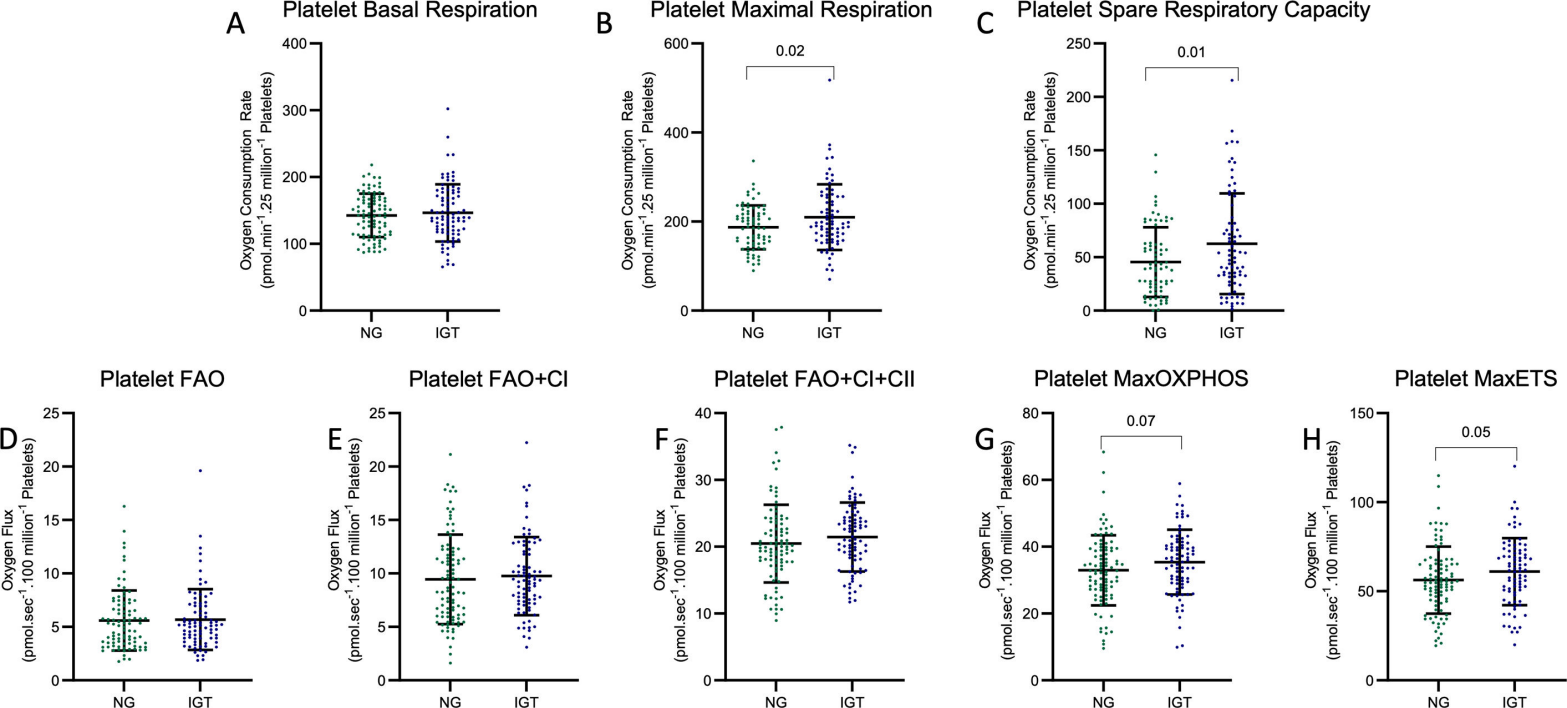
Differences in mitochondrial bioenergetic values between platelets obtained from normoglycemic (NG) and impaired glucose tolerance (IGT) individuals. Significantly higher platelet mitochondrial bioenergetics is observed in maximal respiration, spare respiratory capacities (SRCs), and MaxETS respiration, and higher platelet mitochondrial bioenergetics is observed in MaxOXPHOS. Actual *P* values are shown.

Platelet mitochondrial bioenergetic parameters are presented in Figure 1. Basal respiration, maximal respiration (Max), and SRC are presented in Figure 1A, B and C; leak respiration, ATP-linked respiration, and coupling efficiency are presented in Supplementary Figure S2 A, B, and C. Key parameters were lower in the NG group than in the IGT group. Specifically, average Max was significantly lower in NG participants (187.00 pmol·min^−1^·25 million^−1^ platelets) than IGT participants (210.00 pmol·min^−1^·25 million^−1^ platelets) (*P*=0.02), and average SRC was significantly lower in NG participants (45.42 pmol·min^−1^·25 million^−1^ platelets) than IGT participants (62.57 pmol·min^−1^·25 million^−1^ platelets) (*P*=0.01) (Figure 1B and C). No statistically significant difference was observed in average basal respiration, leak respiration, ATP-linked respiration, and coupling efficiency levels between NG and IGT participants.

Permeabilized platelet respiration was driven by specific substrates providing electrons to specific complexes of the ETS. Outcomes measured were FAO and FAO-dependent respiration, including measures of FAO capacity, FAO + CI, FAO + CI + CII, MaxOXPHOS, and MaxETS (Figure 1D, E, F, G and H). Specifically, we found a non-significant trend of potentially lower average MaxOXPHOS in NG participants (32.94 pmol·sec^−1^·100 million^−1^ platelets) compared with IGT participants (35.39 pmol·sec^−1^·100 million^−1^ platelets) (*P*=0.07), and we found average MaxETS to be significantly lower in NG participants (56.30 pmol·sec^−1^·100 million^−1^ platelets) compared with IGT participants (61.01 pmol·sec^−1^·100 million^−1^ platelets) (*P*=0.05) (Figure 1H and I). No statistically significant differences were observed in average FAO, FAO + CI, and FAO + CI + CII respiration between NG and IGT participants, even though all parameters showed lower average respiration values in the NG group than in the IGT.

To further delineate the differences in mitochondrial bioenergetic parameters in participants closer to their age groups, we separated the participants from both the NG and IGT groups into three age-specific subgroups ranging from 55 to 64 years, 65 to 74 years, and 75 years and above, and analyzed the differences in each age subgroup (Supplementary Table S3). While average values of all the parameters assessed were lower in the NG group than in the IGT group, several parameters were significantly lower in the NG group than in the IGT group in different age subgroups. Specifically, average basal respiration was significantly lower in NG (139.13 pmol·min^−1^·25 million^−1^ platelets) than in IGT participants (160.83 pmol·min^−1^·25 million^−1^ platelets) (*P*=0.03) in the 55 to 64 years subgroup, while it was not significant in the 65 to 74 years and 75 years and above subgroups. Average ATP-linked respiration was significantly lower in NG participants (127.93 pmol·min^−1^·25 million^−1^ platelets) than in IGT participants (146.05 pmol·min^−1^·25 million^−1^ platelets) (*P*=0.04) in the 55 to 64 years subgroup, while it was not significant in the 65 to 74 years and 75 years and above subgroups. Average SRC respiration was significantly lower in NG participants (40.47 pmol·min^−1^·25 million^−1^ platelets) than in IGT participants (62.86 pmol·min^−1^·25 million^−1^ platelets) (*P*=0.02) in the 65 to 74 years subgroup, while it was not significant in the 55 to 64 years and 75 years and above subgroups. Similarly, average FAO + CI + CII, MaxOXPHOS, and MaxETS values were significantly lower in NG (19.47, 31.001, and 53.59 pmol·sec^−1^·100 million^−1^ platelets) than in IGT participants (22.11, 37.15, and 64.12 pmol·sec^−1^·100 million^−1^ platelets) (*P*=0.04, 0.01, and 0.03, respectively) in the 55 to 64 years subgroup, while they were not significant in the 65 to 74 years and 75 years and above.

To further investigate the differences in mitochondrial bioenergetic parameters between NG and PD, NG and D, and PD and D participants, we separated the participants from the IGT group into PD and D subgroups (Supplementary Table S8). We observed significantly higher platelet mitochondrial bioenergetics in the PD group compared with the NG group in maximal respiration (NG=187.00 and PD=210.29, *P*=0.04), SRC (NG=45.42 and PD=59.24, *P*=0.03), and MaxETS respiration (NG=56.30 and PD=65.15, *P*=0.05). No other significant differences were observed between the NG and PD or D groups or in between the PD and D groups.

### Association of platelet mitochondrial bioenergetic parameters with insulin resistance measures and HbA1c

#### Platelet mitochondrial bioenergetic parameters are associated with insulin resistance measures

Spearman correlation coefficients were used to assess relationships between individual platelet mitochondrial bioenergetic parameters and glucose tolerance measures in all the participants combined (NG+IGT) (Table 2, and Supplementary Table S4). Specifically, in intact platelets, significant positive associations were observed between platelet SRC and OGTT_120 (*R*=0.20, *P*=0.02), and OGTT_AUC (*R*=0.17, *P*=0.05), while in permeabilized platelets, significant positive associations were observed between FAO + CI + CII and OGTT_120 (*R*=0.18, *P*=0.03), and OGTT_AUC (*R*=0.18, *P*=0.04), MaxOXPHOS and OGTT_120 (*R*=0.17, *P*=0.04), and MaxETS and OGTT_120 (*R*=0.16, *P*=0.05). Interestingly, the majority of the associations observed were positive.

**Table 2:** Spearman correlation coefficients (R) and *P* values were assessed between platelet (Plt) mitochondrial bioenergetic parameters and OGTT_120 and OGTT_AUC values in all participants combined. *P* values ≤ 0.05 are shown in bold with asterisk (*=≤ 0.05), and *P* values < 0.1 are shown in bold

All plt (NG + IGT)	OGTT_120 (R, P)	OGTT_AUC (R, P)
Plt BASAL	0.03, 0.74	0.02, 0.80
Plt MAX	0.13, 0.13	0.10, 0.26
Plt SRC	0.20*, 0.02	0.17*, 0.05
Plt FAO	0.06, 0.47	0.06, 0.52
Plt FAO + CI	0.10, 0.25	0.10, 0.24
Plt FAO + CI + CII	0.18*, 0.03	0.18*, 0.04
Plt MaxOXPHOS	0.17*, 0.04	0.13, 0.12
Plt MaxETS	0.16*, 0.05	**0.15, 0.08**

To understand the effects of demographic parameters on the associations between platelet mitochondrial bioenergetic parameters and IR measures in all participants combined, we assessed partial correlation coefficients with age, sex, and BMI as the potential confounders.

As shown in Table 3 and Supplementary Table S4, after adjusting for age, SRC remained significantly positively associated with OGTT_120 (*R*=0.18, *P*=0.03) and FAO + CI + CII remained significantly positively associated with OGTT_AUC (*R*=0.16, *P*=0.05). All other associations lost their significance (*P*≥0.05 and ≤ 0.1). After adjusting for sex, associations observed between SRC, FAO + CI + CII, MaxOXPHOS, and MaxETS, and OGTT_120 (*R*=0.20, *P*=0.02; *R*=0.18, *P*=0.03; *R*=0.18, *P*=0.03; and *R*=0.16, *P*=0.05), and SRC, FAO + CI + CII, and OGTT_AUC (*R*=0.17, *P*=0.05; and *R*=0.18, *P*=0.03) remained significant. After adjusting for BMI, SRC remained significantly positively associated with OGTT_120 (*R*=0.19, *P*=0.03), FAO + CI + CII remained significantly positively associated with OGTT_120 (*R*=0.17, *P*=0.04) and OGTT_AUC (*R*=0.17, *P*=0.05), and MaxOXPHOS remained significantly positively associated with OGTT_120 (*R*=0.17, *P*=0.04), while that between MaxETS and OGTT_120 lost significance but showed a potential positive trend (*R*=0.15, *P*=0.06).

**Table 3:** Partial correlation coefficients (R) and *P* values assessed in all participants combined between platelet (Plt) mitochondrial bioenergetic parameters and OGTT_120 and OGTT_AUC values after separate adjustments with age, sex, and BMI, and adjusting for age, sex, and BMI together. *P* values ≤ 0.05 are shown in bold with asterisk (*=≤ 0.05), and *P* values < 0.1 are shown in bold

All plt (NG + IGT)	Age adjusted		Sex adjusted		BMI adjusted		Age, Sex, BMI adjusted	
	OGTT_120 (R, P)	OGTT_AUC (R, P)	OGTT_120 (R, P)	OGTT_AUC (R, P)	OGTT_120 (R, P)	OGTT_AUC (R, P)	OGTT_120 (R, P)	OGTT_AUC (R, P)
Plt BASAL	0.03, 0.69	0.02, 0.78	0.05, 0.58	0.04, 0.68	0.02, 0.84	0.01, 0.93	0.02, 0.78	0.01, 0.90
Plt MAX	0.13, 0.15	0.10, 0.28	**0.15, 0.09**	0.11, 0.21	0.12, 0.17	0.09, 0.35	0.12, 0.19	0.08, 0.39
Plt SRC	0.18*, 0.03	**0.16, 0.07**	0.20*, 0.02	0.17*, 0.05	0.19*, 0.03	**0.15, 0.09**	**0.17, 0.06**	0.14, 0.13
Plt FAO	0.06, 0.51	0.05, 0.55	0.06, 0.47	0.06, 0.52	0.04, 0.63	0.03, 0.76	0.03, 0.72	0.02, 0.83
Plt FAO + CI	0.08, 0.36	0.09, 0.31	0.10, 0.25	0.10, 0.24	0.09, 0.29	0.09, 0.31	0.07, 0.43	0.07, 0.40
Plt FAO + CI + CII	**0.15, 0.06**	0.16*, 0.05	0.18*, 0.03	0.18*, 0.03	0.17*, 0.04	0.17*, 0.05	**0.14, 0.09**	**0.15, 0.09**
Plt MaxOXPHOS	**0.15, 0.07**	0.12, 0.16	0.18*, 0.03	0.14, 0.11	0.17*, 0.04	0.12, 0.16	**0.14, 0.09**	0.11, 0.23
Plt MaxETS	**0.14, 0.09**	0.13, 0.11	0.16*, 0.05	**0.15, 0.08**	**0.15, 0.06**	0.14, 0.11	0.13, 0.12	0.12, 0.16

Further adjusting for age, sex, and BMI together led to loss of all significant associations, even though a trend for potential association remained between SRC and OGTT_120 (*R*=0.17, *P*=0.06).

To examine if the associations observed between platelet mitochondrial bioenergetic parameters and glucose tolerance measures (OGTT_120 and OGTT_AUC values) differed according to glucose tolerance of the participants, we separated the data for NG and IGT groups and assessed the relationships within each group. As shown in Supplementary Table S9, significant associations between platelet mitochondrial bioenergetics and OGTT_120 and OGTT_AUC values were apparent in both the NG and IGT groups. Specifically, in the NG group, significant positive associations were observed between platelet coupling efficiency and OGTT_120 (*R*=0.29, *P*=0.01), and platelet FAO + CI + CII and OGTT_120 (*R*=0.24, *P*=0.02). Again, in the NG group, significant positive associations were observed between platelet coupling efficiency and OGTT_AUC (*R*=0.25, *P*=0.02), platelet FAO + CI and OGTT_AUC (*R*=0.22, *P*=0.04), and platelet FAO + CI + CII and OGTT_AUC (*R*=0.25, *P*=0.02). No significant associations were observed in the IGT group.

We further tested the effects of demographic parameters (age, sex, and BMI) on the associations between platelet mitochondrial bioenergetic parameters and glucose tolerance measures in the NG and IGT participants grouped separately. As shown in Supplementary Table S9, in the NG group, platelet mitochondrial bioenergetic parameters that were previously observed in the unadjusted condition to be significantly associated with OGTT_120 and OGTT_AUC values remained significantly associated after individually adjusting for age, sex, and BMI, and after adjusting for age, sex, and BMI together. Specifically, coupling efficiency remained significantly positively associated with OGTT_120 (*R*=0.30, *P*=0.01 after age adjustment; *R*=0.30, *P*=0.01 after sex adjustment; *R*=0.29, *P*=0.01 after BMI adjustment; and *R*=0.32, *P*=0.01 after adjusting for age, sex, and BMI together), and coupling efficiency remained significantly positively associated with OGTT_AUC (*R*=0.26, *P*=0.02 after age adjustment; *R*=0.26, *P*=0.02 after sex adjustment; *R*=0.25, *P*=0.02 after BMI adjustment; and *R*=0.27, *P*=0.02 after adjusting for age, sex, and BMI together). Similarly, FAO + CI + CII remained significantly positively associated with OGTT_120 (*R*=0.21, *P*=0.05 after age adjustment; *R*=0.25, *P*=0.02 after sex adjustment; *R*=0.24, *P*=0.02 after BMI adjustment; and *R*=0.22, *P*=0.04 after adjusting for age, sex, and BMI together), FAO + CI + CII remained significantly positively associated with OGTT_AUC (*R*=0.23, *P*=0.03 after age adjustment; *R*=0.24, *P*=0.03 after sex adjustment; *R*=0.24, *P*=0.03 after BMI adjustment; and *R*=0.23, *P*=0.04 after adjusting for age, sex, and BMI together). Again, FAO + CI remained significantly positively associated with OGTT_AUC (*R*=0.22, *P*=0.05 after age adjustment; *R*=0.22, *P*=0.04 after sex adjustment; *R*=0.23, *P*=0.04 after BMI adjustment; and *R*=0.22, *P*=0.04 after adjusting for age, sex, and BMI together).

We also tested the associations between platelet mitochondrial bioenergetic parameters and OGTT_120 and OGTT_AUC values in PD and D participants grouped separately (Supplementary Table S10). The participants in these two groups formed the IGT group. We observed a significant negative association between platelet ATP-linked respiration and OGTT_120 after age adjustment (*R*=-0.54, *P*=0.05). No other significant associations were observed in the PD or D group in the unadjusted condition or after adjustments with age, sex, and BMI individually or together.

#### Associations between platelet mitochondrial bioenergetic parameters and HbA1c

Next, we assessed if platelet mitochondrial bioenergetic measures related to HbA1c levels, a measure of historical blood glucose levels. As shown in Supplementary Table S1 and Supplementary Table S5, no significant association was observed by Spearman correlations assessed between platelet mitochondrial bioenergetic parameters and HbA1c. Further adjustments with demographic parameters such as age, sex, and BMI, individually and together, did not affect associations.

### Associations between platelet mitochondrial bioenergetic parameters and historical glucose measures are different based on glucose tolerance

To examine if the associations observed between platelet mitochondrial bioenergetic parameters and HbA1c differed according to glucose tolerance of the participants, we separated the data for NG and IGT groups and assessed the relationships within each group. As shown in Table 4 and Supplementary Table S6, significant positive associationsTable 4 between platelet mitochondrial bioenergetics and HbA1c were apparent in the NG group. Specifically, in the NG group, significant positive associations were observed between SRC and HbA1c (*R*=0.43, *P*=0.01), coupling efficiency and HbA1c (*R*=0.372, *P*=0.02), FAO and HbA1c (*R*=0.38, *P*=0.009), and FAO + CI and HbA1c (*R*=0.39, *P*=0.008), while significant negative association was observed between leak and HbA1c (*R*=-0.311, *P*=0.05). A trend for a potential positive association was observed between Max and HbA1c (*R*=0.32, *P*=0.06). Interestingly, no significant associations were observed in the IGT group.

**Table 4:** Spearman correlation coefficients (R) and *P* values assessed between platelet (Plt) mitochondrial bioenergetic parameters and HbA1c values in NG and IGT participants grouped separately are shown in the first column (unadjusted). Columns 2, 3, 4, and 5 show partial correlation coefficients (R) and *P* values assessed in NG and IGT participants grouped separately between platelet mitochondrial bioenergetic parameters and HbA1c values after individual adjustments with age, sex, and BMI, and after adjusting for age, sex, and BMI together. *P* values ≤ 0.05 are shown in bold with an asterisk (*=≤ 0.05, **=≤ 0.01), and *P* values < 0.1 are shown in bold

	HbA1c									
NG and IGT plt separated	Unadjusted		Age adjusted		Sex adjusted		BMI adjusted		Age, Sex, BMI adjusted	
	NG (R, P)	IGT (R, P)	NG (R, P)	IGT (R, P)	NG (R, P)	IGT (R, P)	NG (R, P)	IGT (R, P)	NG (R, P)	IGT (R, P)
Plt BASAL	0.02, 0.92	0.07, 0.61	0.01, 0.97	0.02, 0.90	-0.01, 0.95	0.01, 0.93	-0.06, 0.68	0.06, 0.65	-0.10, 0.55	-0.05, 0.73
Plt MAX	**0.32, 0.06**	0.02, 0.92	**0.30, 0.08**	-0.03, 0.87	**0.32, 0.06**	-0.03, 0.87	**0.28, 0.10**	-0.03, 0.83	0.28, 0.12	-0.13, 0.38
Plt SRC	0.43*, 0.01	-0.03, 0.82	0.41*, 0.01	-0.05, 0.75	0.43*, 0.01	-0.04, 0.79	0.41*, 0.01	-0.10, 0.49	0.41*, 0.02	-0.13, 0.39
Plt FAO	0.38**, 0.009	0.01, 0.95	0.37*, 0.01	0.01, 0.94	0.39*, 0.01	0.04, 0.79	0.40**, 0.008	-0.06, 0.65	0.39*, 0.01	-0.04, 0.78
Plt FAO + CI	0.39**, 0.008	-0.07, 0.64	0.37*, 0.01	-0.03, 0.86	0.39**, 0.007	-0.06, 0.67	0.37*, 0.01	-0.09, 0.55	0.37*, 0.01	-0.06, 0.67
Plt FAO + CI + CII	**0.24, 0.09**	-0.14, 0.34	0.23, 0.12	-0.10, 0.48	**0.25, 0.09**	-0.13, 0.36	0.19, 0.21	-0.11, 0.47	0.18, 0.25	-0.08, 0.57
Plt MaxOXPHOS	0.08, 0.62	-0.14, 0.30	0.06, 0.68	-0.13, 0.36	0.08, 0.61	-0.14, 0.32	0.03, 0.87	-0.15, 0.29	0.02, 0.91	-0.14, 0.35
Plt MaxETS	0.23, 0.11	-0.12, 0.38	0.22, 0.14	-0.11, 0.45	0.24, 0.11	0.12, 0.39	0.20, 0.17	-0.12, 0.39	0.20, 0.19	-0.11, 0.44

We further tested the effects of demographic parameters such as age, sex, and BMI on the associations between platelet mitochondrial bioenergetic parameters and historical glucose measure in the NG and IGT participants grouped separately. As shown in Table 4 and Supplementary Table S6, in the NG group, platelet mitochondrial bioenergetic parameters that were previously observed in unadjusted condition to be significantly associated with HbA1c remained significantly associated after individually adjusting for age, sex, and BMI. Specifically, SRC remained significantly positively associated with HbA1c (*R*=0.41, *P*=0.01 after age adjustment; *R*=0.43, *P*=0.01 after sex adjustment; *R*=0.41, *P*=0.01 after BMI adjustment; and *R*=0.41, *P*=0.02 after adjusting for age, sex, and BMI together), leak remained significantly negatively associated with HbA1c (*R*=-0.311, *P*=0.05 after age adjustment; *R*=-0.356, *P*=0.02 after sex adjustment; *R*=-0.342, *P*=0.03 after BMI adjustment; and *R*=-0.381, *P*=0.02 after adjusting for age, sex, and BMI together), and coupling efficiency remained significantly positively associated with HbA1c (*R*=0.37, *P*=0.01 after age adjustment; *R*=0.395, *P*=0.01 after sex adjustment; *R*=0.355, *P*=0.03 after BMI adjustment; and *R*=0.374, *P*=0.02 after adjusting for age, sex, and BMI together). Similarly, FAO remained significantly positively associated with HbA1c (*R*=0.37, *P*=0.01 after age adjustment; *R*=0.39, *P*=0.01 after sex adjustment; *R*=0.40, *P*=0.008 after BMI adjustment; and *R*=0.39, *P*=0.01 after adjusting for age, sex, and BMI together), and FAO + CI remained significantly positively associated with HbA1c (*R*=0.37, *P*=0.01 after age adjustment; *R*=0.39, *P*=0.007 after sex adjustment; and *R*=0.37, *P*=0.01 after BMI adjustment; and *R*=0.37, *P*=0.01 after adjusting for age, sex, and BMI together). Overall, the NG group showed a positive directionality, while the IGT group showed no association.

We also tested the associations between platelet mitochondrial bioenergetic parameters and HbA1c values in PD and D participants grouped separately (Supplementary Table S11). The participants in these two groups formed the IGT group. We observed significant positive associations in unadjusted condition in the PD group between platelet basal respiration and ATP-linked respiration and OGTT_120 (*R*=0.40, *P*=0.02; and *R*=0.38, *P*=0.03). The positive association between basal respiration and HbA1c remained significant after sex and BMI adjustments (*R*=0.35, *P*=0.05; and *R*=0.36, *P*=0.04), and the positive association between ATP-linked respiration and HbA1c remained significant after BMI adjustment (*R*=0.35, *P*=0.05). These associations were lost after individual adjustment with age, and after adjusting for age, sex, and BMI together. In the D group, we observed a significant negative association between platelet leak respiration and HbA1c after sex adjustment (*R*=-0.44, *P*=0.05). No other significant associations were observed in the PD or D group before adjustments or after adjustments with age, sex, and BMI individually or together.

## Discussion

Studies have shown that platelets are suitable for *ex vivo* analysis of human mitochondria with excellent reproducibility and stability [13]. In this study, we compared mitochondrial function of platelets collected from NG and hyperglycemic (IGT) individuals and observed a higher level of mitochondrial bioenergetics in hyperglycemic individuals. When the IGT group was divided into PD and D groups, we observed higher levels of mitochondrial bioenergetics in the PD individuals compared with the NG individuals. Similar increases in mitochondrial respiration were previously observed in another type of circulating blood cell, peripheral blood mononuclear cells (PBMCs), collected from individuals with diabetes [14], indicating that circulating cells may exhibit systemic alterations associated with IR and T2DM. IR and T2DM are known to cause platelet hyperactivity in obesity and hypertension [15]. Platelet activation is driven by higher mitochondrial functioning in aging [16], and activated platelets from hypertension patients show higher mitochondrial maximal respiration and reserve capacities [17], similar to our findings. It is important to note that platelets are highly prone to activation during handling, which may seem like an apparent reason behind their hyperactivity in our study. However, we used PGE1ɑ to prevent platelet activation [18] during their isolation from whole blood, which indicates that the increase in mitochondrial bioenergetic functions in IGT platelets reported in this study may be due to hyperactivity caused by IGT. Similar increases in mitochondrial maximal respiration and SRC were also observed in adipocytes obtained from metabolically unhealthy obese individuals who showed whole-body IR, subclinical inflammation, and hepatic steatosis, as compared with metabolically healthy obese individuals [19].

Using the second approach that utilizes fatty acid and glucose-metabolism-derived substrates, we observed a significantly higher maximal uncoupled FAO-mediated respiration (MaxETS) in platelets obtained from IGT individuals. One study conducted in PBMCs showed that increased total and uncoupled mitochondrial oxygen consumption in diabetes is associated with ROS generation by ETC components and other enzymatic sites and correlated with endothelial dysfunction [14]. T2DM is generally associated with higher levels of lipoproteins and triglycerides in plasma and an increase in utilization of free fatty acids [20]. In another study, platelets from T2DM individuals who developed coronary in-stent restenosis were found to exhibit mitochondrial bioenergetic alteration specified by dependence on FAO [21].

Our results showed significant positive associations between measures of mitochondrial bioenergetics and IR when data from all the participants were included in the analysis (NG + IGT). We further tested the relationship between platelet mitochondrial bioenergetics and glucose measures in the NG and IGT groups separately and observed significant associations in the NG group, while no observed associations were present in the IGT group. Further separating the participants comprising the IGT group into PD and D showed no significant associations with glucose measures.

Overall, our findings reiterate the suggestions that increased mitochondrial respiration in cells from insulin-resistant individuals may indicate a compensatory coping mechanism against IR and its pitfalls. Studies have shown that in prediabetes and early-stage T2DM, mitochondrial complexes I and II dependent OXPHOS increased greatly and then declined at late-stage T2DM in the liver but not skeletal muscles, indicating an adaptive mechanism occurring in liver tissue, a primary driver of T2DM [7,22]. It is important to note that approximately 72% of our IGT group (69 out of 96) were older adults with prediabetes, and approximately 28% (27 out of 96) were older adults with diabetes. This may cause our IGT group to behave similarly to a group of individuals who were living with prediabetes and early-stage diabetes rather than late-stage diabetes. It is important to note that when the IGT group was divided into PD and D groups, we observed higher levels of mitochondrial bioenergetics in the PD individuals compared with the NG individuals, while there were no differences between the D and NG individuals.

Our data, therefore, demonstrate that the difference in platelet mitochondrial function between NG and IGT groups is dependent on the physiological parameters defining these groups. These findings also suggest better glycemic control in platelets from NG older adults than IGT, suggesting that circulating platelets may closely represent tissues that are primarily involved in aggravating pathologies due to IR. Mean platelet volume (MPV) is a biological marker of platelet function and activity [23]. MPV was found to be higher in newly diagnosed T2DM patients and individuals at the early stages of diabetes as compared with NG individuals, indicating that PD individuals also have higher platelet reactivity than NGs. The same study also demonstrated that higher MPV associates with increased platelet activity and higher risk of vascular complications and cardiovascular disease [24]. While MPV was robustly associated with diabetes, platelet function, measured by platelet aggregation, adhesion, and agglutination, was not consistently associated [25]. Opposite findings for platelet function were also reported by another group, who showed that in participants with diabetes mellitus, but without cardiovascular disease, platelets were not hyperresponsive and platelet aggregation was not associated with glycemic status [26]. Previous studies have shown that with energetic stress associated with high-fat diets, there is increased mitochondrial content along with IR [27]. Rats fed with high-fat diets show IR and an associated gradual increase in mitochondrial content in their skeletal muscles [28]. Mitochondria are at the interphase of impaired fuel utilization, including impaired FAO, lipotoxicity, and IR, thereby playing a pivotal role in oxidative metabolism [29]. It was previously suggested that bone marrow and platelets of type 2 diabetic individuals are exposed to increased mitochondrial oxidative stress, and this stress signature is associated with decreased mitochondrial energy production [30]. Our results may therefore imply an IR-mediated stress signature in platelets obtained from individuals with prediabetes and diabetes and may be associated with increased mitochondrial oxidative stress observed in hyperglycemia [5].

Glycation of proteins is observed in prediabetes and diabetes, causing altered functions [31]. Altered functioning of platelet mitochondrial proteins due to diabetes-related glycation [32] may be the reason why the IGT group shows higher average platelet respiration and lack of associations with glucose measures. Platelets exposed to high glucose concentrations mimicking diabetes have shown increased mitochondrial respiration, which may indicate an adaptation to high availability of glucose. Reducing mitochondrial respiration with metformin in this case did not inhibit activation of platelets [32]. One study on the molecular mechanisms mediating mitochondrial dysfunction and damage in platelets of diabetic subjects found that this is mediated by phosphorylation on serine 15 and subsequent activation of p53 through activities of the aldose reductase protein. While mild dysfunction and damage to mitochondria lead to hyperactivity, severe damage leads to apoptosis [33]. Our findings, therefore, uncover an abnormal pattern of platelet mitochondrial functions in IGT individuals as compared with NG, enhancing our current understanding of systemic abnormalities in the context of IR, hyperglycemia, and T2DM. Further work is needed to test whether the different levels of mitochondrial bioenergetics observed in the NG and IGT groups in our study and altered associations between mitochondrial bioenergetics and glucose measures in these groups are due to differences in platelet activities, mitochondrial content, oxidative stress, protein glycation, or any other mechanism.

## Strengths and limitations

The major strength of this study lies in the utilization of a well-characterized cohort with two complementary respirometric protocols that provide a detailed assessment of mitochondrial bioenergetics in platelets that are highly metabolically active circulating cells. This study demonstrates that platelet mitochondrial respiratory capacity may be used as an indicator for systemic metabolic dysfunction in hyperglycemia. The major limitation of this study is the lower number of participants with diabetes compared with participants with prediabetes in the IGT group (69 and 27, respectively), which may have reduced the impact of hyperglycemia-mediated pathological alterations on platelet mitochondria. Another limitation of the study is the lack of traditional measurements of platelet function, including platelet aggregation, platelet adhesion, and platelet agglutination, using techniques like light transmission aggregometry and flow cytometry, and alternative measurements like MPV, to determine alterations in platelet function due to hyperglycemia and how they are related to mitochondrial bioenergetics.

## Conclusions

Platelets are easily accessible and highly metabolically active cells that may be studied in relation to hyperglycemia to assess the emerging mitochondria-targeted anti-diabetic therapeutics. We identify mitochondrial bioenergetic parameters in platelets that differ between NG and hyperglycemic older adults and reveal stress signatures associated with glucose tolerance measures. IR and T2DM are major risk factors for age-related disorders, and systemic mitochondrial bioenergetic capacities can report on pathological mechanisms associated with hyperglycemia in highly metabolically active tissues. Higher platelet mitochondrial bioenergetic functions may imply a compensatory coping mechanism to IR. Further work is needed to elucidate the molecular factors mediating mitochondrial bioenergetic differences and stress signatures in platelets.

**Clinical Perspectives**

- Older adults with impaired glucose tolerance (IGT), insulin resistance (IR), and type-2 diabetes mellitus (T2DM) experience a higher risk for age-related disorders.
- Platelet mitochondrial bioenergetic capacities are altered in older individuals with IGT and IR as compared with normoglycemic older adults and associate with the hallmarks of T2DM.
- Platelet mitochondrial bioenergetics are affected by metabolic disorders such as IGT and IR and reveal stress signatures associated with glucose tolerance measures.

## Supplementary material

online supplementary material 1.

online supplementary figure 1.

online supplementary figure 2.

## Data Availability

To request access to data, please contact Wake Forest ADRC through the WakeSHARE link: https://www.wakeshare.org/

## Abbreviations

A/R: antimycin A + rotenone
ATP: adenosine triphosphate
BMI: body mass index
CIV: complex IV
D: diabetic
ETC: electron transport chain
ETS: electron transport system
FAO: fatty acid oxidation
FCCP: carbonyl cyanide-4-(trifluoromethoxy) phenylhydrazone
HbA1c: hemoglobin A1
IGT: impaired glucose tolerance
MPV: mean platelet volume
Max: maximal respiration
MaxETS: maximal electron transport system
MaxOXPHOS: fatty acid oxidation (FAO)-dependent maximal coupled respiration
NG: normoglycemic
OCR: oxygen consumption rate
OGTT: oral glucose tolerance test
OGTT_120: oral glucose tolerance test values measured 120 min after glucose administration
OGTT_AUC: oral glucose tolerance test area under the curve
OXPHOS: oxidative phosphorylation
PBMC: peripheral blood mononuclear cell
PD: prediabetics
PGE1: prostaglandin E1
Plt: Platelets
ROS: reactive oxygen species
SRC: spare respiratory capacity
T2DM: type-2 diabetes mellitus
TMPD: N,N,N′,N′-tetramethyl-p-phenylenediamine
XF: extracellular flux
