# Supplementary material for: Platelets from older adults exhibit differences in mitochondrial function associated with impaired glucose metabolism
Source: Clin Sci (Lond). 2026 Jan 14;140(1):65–79. doi: 10.1042/CS20242841 (PMC12862959; doi:10.1042/CS20242841)
Supplement: online supplementary material 1. [file cs-140-1-CS20242841-s001.docx]

| **ALL PLT (NG + IGT)** | **HbA1c** | | | | |
| --- | --- | --- | --- | --- | --- |
|  | **Unadjusted (R, P)** | **Age adjusted (R, P)** | **Sex adjusted (R, P)** | **BMI adjusted (R, P)** | **Age, Sex, BMI adjusted (R, P)** |
| **Plt BASAL** | 0.07, 0.48 | 0.07, 0.51 | 0.03, 0.74 | 0.05, 0.65 | 0.001, 0.99 |
| **Plt MAX** | 0.13, 0.25 | 0.13, 0.26 | 0.11, 0.32 | 0.08, 0.45 | 0.06, 0.58 |
| **Plt SRC** | 0.10, 0.38 | 0.11, 0.34 | 0.10, 0.38 | 0.04, 0.70 | 0.05, 0.67 |
| **Plt FAO** | 0.09, 0.40 | 0.09, 0.38 | 0.10, 0.32 | 0.05, 0.62 | 0.07, 0.51 |
| **Plt FAO+CI** | 0.08, 0.41 | 0.09, 0.37 | 0.09, 0.36 | 0.06, 0.54 | 0.08, 0.46 |
| **Plt FAO+CI+CII** | 0.04, 0.66 | 0.05, 0.62 | 0.05, 0.62 | 0.02, 0.84 | 0.03, 0.78 |
| **Plt MaxOXPHOS** | 0.02, 0.82 | 0.03, 0.79 | 0.03, 0.77 | -0.01, 0.91 | -0.003, 0.98 |
| **Plt MaxETS** | 0.07, 0.47 | 0.08, 0.45 | 0.08, 0.43 | 0.05, 0.61 | 0.06, 0.54 |

Supplemental Table 1: Spearman correlation coefficients (R) and P values assessed between platelet mitochondrial bioenergetic parameters and HbA1c values in all participants combined are shown in the first column (unadjusted). Columns 2, 3, 4, and 5 show partial correlation coefficients (R) and P values assessed in all participants combined between platelet mitochondrial bioenergetic parameters and HbA1c values after individual adjustments with age, sex, and BMI, and after adjusting for age, sex, and BMI together.

| **DEMOGRAPHICS AND GLUCOSE TOLERANCE MEASURES** | **Normoglycemic** | | **Impaired Glucose Tolerance** | | **P values** | **Normoglycemic** | | **Impaired Glucose Tolerance** | | **P values** | **Normoglycemic** | | **Impaired Glucose Tolerance** | | | **P values** | |
| --- | --- | --- | --- | --- | --- | --- | --- | --- | --- | --- | --- | --- | --- | --- | --- | --- | --- |
|  | **55 to 64 years = 42 Participants** | | **55 to 64 years = 29 Participants (25PD+4D)** | |  | **65 to 74 years = 46 Participants** | | **65 to 74 years = 48 Participants (31PD+17D)** | |  | **75 years and above = 24 Participants** | | **75 years and above = 17 Participants (12PD+5D)** | | |  |  |
|  | **Average** | **Std Dev** | **Average** | **Std Dev** |  | **Average** | **Std Dev** | **Average** | **Std Dev** |  | **Average** | **Std Dev** | **Average** | **Std Dev** |  | |  |
| **Age (years)** | 59.88 | 2.72 | 64.35 | 2.47 | **0.02*** | 68.98 | 2.94 | 69.42 | 2.71 | 0.45 | 80.13 | 4.72 | 80.74 | 5.21 | 0.70 | |  |
| **BMI (kg/m^2^)** | 25.01 | 5.23 | 30.39 | 6.51 | **0.001*** | 25.91 | 6.26 | 27.94 | 5.50 | **0.10** | 24.12 | 3.90 | 27.54 | 4.88 | **0.02*** | |  |
| **OGTT_120** | 97.57 | 21.39 | 162.59 | 31.46 | **2.992E-09*** | 109.48 | 22.49 | 175.34 | 26.25 | **8.982E-17*** | 118.38 | 18.39 | 175.74 | 29.88 | **9.715E-07*** | |  |
| **OGTT_AUC** | 243.11 | 35.29 | 308.03 | 35.70 | **1.382E-07*** | 254.97 | 30.32 | 335.05 | 40.00 | **2.968E-12*** | 265.04 | 22.37 | 320.53 | 37.03 | **5.574E-0.5*** | |  |
| **HbA1c** | 5.47 | 0.31 | 5.96 | 0.56 | **0.002*** | 5.42 | 0.29 | 5.95 | 0.64 | **3.459E-05*** | 5.35 | 0.14 | 5.73 | 0.49 | **0.03*** | |  |

Supplemental Table 2: Participant demographics and glucose measures separated by age sub-groups, 55 to 64 years, 65 to 74 years, 75 years and above. Abbreviations: BMI – Body Mass Index; OGTT_120 – Oral Glucose Tolerance Test 120 minutes after glucose administration, OGTT_AUC – Oral Glucose Tolerance Test Area Under The Curve, HbA1c – Hemoglobin A1c. Values are Mean ± Standard Deviation. P values ≤ 0.05 are shown in bold with asterisk. * = ≤ 0.05

|  | **Normoglycemic** | | **Impaired Glucose Tolerance** | | **P values** | **Normoglycemic** | | **Impaired Glucose Tolerance** | | **P values** | **Normoglycemic** | | **Impaired Glucose Tolerance** | | | **P values** | |
| --- | --- | --- | --- | --- | --- | --- | --- | --- | --- | --- | --- | --- | --- | --- | --- | --- | --- |
|  | **55 to 64 years = 42 Participants** | | **55 to 64 years = 29 Participants (25PD+4D)** | |  | **64 to 74 years = 46 Participants** | | **64 to 74 years = 48 Participants (31PD+17D)** | |  | **75 years and above = 24 Participants** | | **75 years and above = 17 Participants (12PD+5D)** | | |  |  |
|  | **Average** | **Std Dev** | **Average** | **Std Dev** |  | **Average** | **Std Dev** | **Average** | **Std Dev** |  | **Average** | **Std Dev** | **Average** | **Std Dev** |  | |  |
| **Plt BASAL** | 139.13 | 31.92 | 160.83 | 40.00 | **0.03*** | 144.30 | 30.95 | 140.61 | 44.47 | 0.67 | 145.82 | 35.39 | 13742 | 34.28 | 0.51 | |  |
| **Plt LEAK** | 11.55 | 6.10 | 15.56 | 12.61 | 0.15 | 11.12 | 5.73 | 11.59 | 9.94 | 0.79 | 11.83 | 5.23 | 10.32 | 4.84 | 0.44 | |  |
| **Plt MAX** | 178.58 | 58.00 | 208.02 | 76.00 | **0.10** | 173.27 | 47.85 | 194.64 | 80.79 | 0.14 | 183.49 | 39.09 | 190.19 | 61.38 | 0.73 | |  |
| **Plt SRC** | 47.88 | 36.98 | 65.05 | 47.65 | 0.17 | 40.47 | 26.79 | 62.86 | 49.39 | **0.02*** | 49.74 | 30.64 | 57.86 | 36.82 | 0.54 | |  |
| **Plt ATP-linked** | 127.93 | 28.65 | 146.05 | 37.05 | **0.04*** | 133.98 | 27.59 | 139.06 | 69.89 | 0.66 | 136.18 | 30.32 | 127.81 | 31.63 | 0.46 | |  |
| **Plt COUPLING EFFICIENCY** | 92.18 | 3.98 | 91.10 | 7.24 | 0.49 | 93.12 | 4.08 | 93.36 | 8.24 | 0.86 | 93.99 | 4.06 | 92.97 | 4.29 | 0.50 | |  |
| **Plt FAO** | 5.34 | 2.89 | 5.63 | 2.23 | 0.68 | 5.44 | 2.25 | 5.54 | 3.27 | 0.86 | 6.35 | 3.38 | 6.15 | 2.31 | 0.84 | |  |
| **Plt FAO+CI** | 8.81 | 4.11 | 9.82 | 3.21 | 0.29 | 9.41 | 3.75 | 9.26 | 3.83 | 0.86 | 10.72 | 4.73 | 11.00 | 3.42 | 0.90 | |  |
| **Plt FAO+CI+CII** | 19.47 | 5.69 | 22.11 | 4.28 | **0.04*** | 20.48 | 5.21 | 20.54 | 5.33 | 0.96 | 22.39 | 6.52 | 22.76 | 5.33 | 0.86 | |  |
| **Plt MaxOXPHOS** | 31.001 | 9.18 | 37.15 | 9.36 | **0.01*** | 33.05 | 9.72 | 34.06 | 9.70 | 0.64 | 36.37 | 12.98 | 36.24 | 9.50 | 0.97 | |  |
| **Plt MaxETS** | 53.59 | 18.47 | 64.12 | 17.05 | **0.03*** | 55.14 | 16.35 | 58.09 | 19.48 | 0.46 | 63.74 | 21.45 | 63.99 | 17.50 | 0.97 | |  |

Supplemental Table 3: Mitochondrial bioenergetic parameters separated by age sub-groups, 55 to 64 years, 65 to 74 years, 75 years and above. P values ≤ 0.05 are shown in bold with asterisk. * = ≤ 0.05

|  | **ALL PLT (NG + IGT) (Unadjusted)** | | **ALL PLT (NG + IGT) Age adjusted** | | **ALL PLT (NG + IGT) Sex adjusted** | | **ALL PLT (NG + IGT) BMI adjusted** | | **ALL PLT (NG+IGT) Age, Sex, BMI adjusted** | |
| --- | --- | --- | --- | --- | --- | --- | --- | --- | --- | --- |
|  | **OGTT_120 (R, P)** | **OGTT_AUC (R, P)** | **OGTT_120 (R, P)** | **OGTT_AUC (R, P)** | **OGTT_120 (R, P)** | **OGTT_AUC (R, P)** | **OGTT_120 (R, P)** | **OGTT_AUC (R, P)** | **OGTT_120 (R, P)** | **OGTT_AUC (R, P)** |
| **Plt LEAK** | -0.002, 0.983 | -0.041, 0.636 | 0.012, 0.887 | -0.035, 0.693 | -0.007, 0.938 | -0.041, 0.642 | -0.010, 0.909 | -0.054, 0.538 | -0.008, 0.928 | -0.051, 0.565 |
| **Plt ATPlinked** | 0.040, 0.628 | 0.037, 0.657 | 0.039, 0.633 | 0.036, 0.673 | 0.055, 0.502 | 0.050, 0.558 | 0.031, 0.708 | 0.026, 0.761 | 0.031, 0.706 | 0.025, 0.765 |
| **Plt Coupling Efficiency** | 0.064, 0.453 | 0.108, 0.215 | 0.043, 0.612 | 0.097, 0.268 | 0.068, 0.422 | 0.108, 0.216 | 0.066, 0.440 | 0.111, 0.204 | 0.054, 0.530 | 0.102, 0.246 |

Supplemental Table 4: Spearman correlation coefficients (R) and P values assessed between platelet leak respiration, ATPlinked respiration, and coupling efficiency and OGTT_120 and OGTT_AUC values in all participants combined are shown in the first column (unadjusted). Columns 2, 3, 4, and 5 show partial correlation coefficients (R) and P values assessed in all participants combined between platelet mitochondrial bioenergetic parameters and OGTT_120 and OGTT_AUC values after individual adjustments with age, sex, and BMI, and after adjusting for age, sex, and BMI together.

| **ALL PLT (NG + IGT)** | **HbA1c** | | | | |
| --- | --- | --- | --- | --- | --- |
|  | **Unadjusted (R, P)** | **Age adjusted (R, P)** | **Sex adjusted (R, P)** | **BMI adjusted (R, P)** | **Age, Sex, BMI adjusted (R, P)** |
| **Plt LEAK** | -0.052, 0.623 | -0.061, 0.563 | -0.075, 0.478 | -0.069, 0.516 | -0.109, 0.309 |
| **Plt ATPlinked** | 0.087, 0.393 | 0.085, 0.409 | 0.053, 0.606 | 0.064, 0.533 | 0.028, 0.790 |
| **Plt Coupling Efficiency** | 0.085, 0.417 | 0.095, 0.369 | 0.097, 0.356 | 0.075, 0.477 | 0.112, 0.297 |

Supplemental Table 5: Spearman correlation coefficients (R) and P values assessed between platelet leak respiration, ATPlinked respiration, and coupling efficiency and HbA1c values in all participants combined are shown in the first column (unadjusted). Columns 2, 3, 4, and 5 show partial correlation coefficients (R) and P values assessed in all participants combined between platelet mitochondrial bioenergetic parameters and HbA1c values after individual adjustments with age, sex, and BMI, and after adjusting for age, sex, and BMI together.

|  | **HbA1c** | | | | | | | | | |
| --- | --- | --- | --- | --- | --- | --- | --- | --- | --- | --- |
|  | **Unadjusted** | | **Age adjusted** | | **Sex adjusted** | | **BMI adjusted** | | **Age, Sex, BMI adjusted** | |
|  | **NG (R, P)** | **IGT (R, P)** | **NG (R, P)** | **IGT (R, P)** | **NG (R, P)** | **IGT (R, P)** | **NG (R, P)** | **IGT (R, P)** | **NG (R, P)** | **IGT (R, P)** |
| **Plt LEAK** | **-0.311*, 0.05** | -0.041, 0.77 | **-0.311*, 0.05** | -0.088, 0.53 | **-0.356*, 0.02** | -0.065, 0.65 | **-0.342*, 0.03** | -0.065, 0.65 | **-0.381*, 0.02** | -0.162, 0.27 |
| **Plt ATPlinked** | 0.107, 0.49 | 0.081, 0.56 | 0.093, 0.55 | 0.038, 0.79 | 0.087, 0.58 | 0.029, 0.84 | 0.024, 0.88 | 0.081, 0.57 | -0.018, 0.91 | -0.010, 0.95 |
| **Plt Coupling Efficiency** | **0.372*, 0.02** | 0.073, 0.60 | **0.37*, 0.02** | 0.105, 0.46 | **0.395*, 0.01** | 0.087, 0.54 | **0.355*, 0.03** | 0.065, 0.65 | **0.374*, 0.02** | 0.152, 0.30 |

Supplemental Table 6: Spearman correlation coefficients (R) and P values assessed between platelet leak respiration, ATPlinked respiration, and coupling efficiency and HbA1c values in NG and IGT participants grouped separately are shown in the first column (unadjusted). Columns 2, 3, 4, and 5 show partial correlation coefficients (R) and P values assessed in NG and IGT participants grouped separately between platelet leak respiration, ATPlinked respiration, and coupling efficiency and HbA1c values after individual adjustments with age, sex, and BMI, and after adjusting for age, sex, and BMI together.

| **DEMOGRAPHICS AND GLUCOSE TOLERANCE MEASURES** | **Normoglycemics (NG = 112)** | | | | **Impaired Glucose Tolerance (N = 96)** | | | | **Pre-Diabetics (PD = 69)** | | | | | **Diabetics (D = 27)** | | | | | **P values** | | | |
| --- | --- | --- | --- | --- | --- | --- | --- | --- | --- | --- | --- | --- | --- | --- | --- | --- | --- | --- | --- | --- | --- | --- |
|  | **Max** | **Min** | **Average** | **Std Dev** | **Max** | **Min** | **Average** | **Std Dev** | **Max** | **Min** | **Average** | **Std Dev** | **Max** | | **Min** | **Average** | **Std Dev** | **NG vs IGT** | | **NG vs PD** | **NG vs D** | **PD vs D** |
| **Age (years)** | 95.00 | 55.00 | 67.96 | 8.23 | 92.00 | 56.00 | 69.22 | 7.48 | 92.00 | 56.00 | 68.38 | 7.42 | 87.00 | | 56.00 | 70.40 | 7.40 | 0.25 | | 0.85 | **0.02*** | **0.04*** |
| **BMI (kg/m^2^)** | 46.90 | 18.20 | 27.03 | 5.13 | 48.30 | 17.40 | 28.61 | 5.84 | 44.40 | 17.40 | 28.29 | 5.49 | 48.30 | | 20.00 | 29.07 | 6.29 | **0.04*** | | **0.0004*** | **0.005*** | 0.38 |
| **ogtt_120** | 139.00 | 58.50 | 107.07 | 22.69 | 255.00 | 100.50 | 171.63 | 29.34 | 192.50 | 140.30 | 161.88 | 15.69 | 255.00 | | 100.50 | 204.56 | 39.11 | **1.638E-30*** | | **1.39855E-32*** | **4.79553E-16*** | **2.71133E-10*** |
| **ogtt_AUC** | 335.78 | 161.11 | 253.04 | 31.78 | 421.06 | 245.06 | 323.38 | 39.80 | 408.05 | 245.06 | 310.57 | 32.61 | 421.06 | | 330.08 | 370.05 | 26.55 | **8.168E-22*** | | **3.03635E-17*** | **1.77084E-11*** | **4.21643E-07*** |
| **HbA1c** | 6.30 | 4.90 | 5.47 | 0.31 | 7.90 | 4.70 | 5.88 | 0.62 | 6.20 | 4.70 | 5.66 | 0.30 | 7.90 | | 4.90 | 6.11 | 0.76 | **2.207E-06*** | | **0.0001*** | **0.0004*** | **0.005*** |
| **Sex** | Female | Male | Female% | Male% | Female | Male | Female% | Male% | Female | Male | Female% | Male% | Female | | Male | Female% | Male% |  | |  |  |  |
|  | 85 | 27 | 76% | 24% | 76 | 20 | 79% | 21% | 57 | 12 | 82.6% | 17.4% | 19 | | 8 | 70.4% | 29.6% |  | |  |  |  |

Supplemental Table 7: Participant demographics and glucose measures. Participants are grouped into PD and D participants who comprise the IGT group. Abbreviations: BMI – Body Mass Index; OGTT_120 – Oral Glucose Tolerance Test 120 minutes after glucose administration, OGTT_AUC – Oral Glucose Tolerance Test Area Under The Curve, HbA1c – Hemoglobin A1c. Values are Mean ± Standard Deviation. P values ≤ 0.05 are shown in bold with asterisk. * = ≤ 0.05

| **PD and D Separated** | **Normoglycemics (NG = 112)** | | | | **Pre-Diabetics (PD = 69)** | | | | **Diabetics (D = 27)** | | | | **P values** | | |
| --- | --- | --- | --- | --- | --- | --- | --- | --- | --- | --- | --- | --- | --- | --- | --- |
|  | **Max** | **Min** | **Average** | **Std Dev** | **Max** | **Min** | **Average** | **Std Dev** | **Max** | **Min** | **Average** | **Std Dev** | **NG vs PD** | **NG vs D** | **PD vs D** |
| **Plt BASAL** | 218.16 | 87.24 | 142.60 | 32.38 | 302.22 | 68.84 | 151.05 | 43.75 | 259.90 | 65.45 | 139.30 | 39.68 | 0.37 | 0.83 | 0.31 |
| **Plt MAX** | 336.32 | 85.37 | 187.00 | 50.69 | 517.78 | 48.41 | 210.29 | 81.89 | 372.17 | 56.86 | 179.37 | 63.01 | **0.04*** | 0.45 | 0.22 |
| **Plt SRC** | 145.64 | 0.12 | 45.42 | 36.82 | 215.55 | 1.60 | 59.24 | 58.81 | 131.74 | 6.54 | 40.07 | 38.65 | **0.03*** | 0.18 | 0.33 |
| **Plt LEAK** | 27.45 | 0.61 | 11.42 | 5.81 | 70.82 | 0.03 | 12.15 | 11.12 | 54.07 | 0.47 | 13.36 | 9.21 | 0.46 | 0.54 | 0.78 |
| **Plt ATP-linked** | 203.93 | 81.72 | 132.07 | 28.77 | 532.57 | 64.95 | 147.24 | 65.60 | 205.84 | 59.41 | 126.89 | 33.60 | 0.20 | 0.68 | 0.11 |
| **Plt Coupling Efficiency** | 99.42 | 81.53 | 92.29 | 3.39 | 99.98 | 61.84 | 92.38 | 5.65 | 99.55 | 79.20 | 90.81 | 4.48 | 0.63 | 0.46 | 0.48 |
| **Plt FAO** | 16.27 | 1.76 | 5.59 | 2.79 | 19.61 | 1.92 | 6.04 | 3.22 | 11.17 | 1.86 | 5.18 | 2.05 | 0.79 | 1.00 | 0.51 |
| **Plt FAO+CI** | 21.14 | 1.62 | 9.45 | 4.16 | 22.24 | 3.09 | 10.24 | 3.96 | 18.24 | 4.09 | 9.08 | 3.00 | 0.51 | 0.97 | 0.36 |
| **Plt FAO+CI+CII** | 37.88 | 8.95 | 20.48 | 5.78 | 35.18 | 11.70 | 22.39 | 5.29 | 34.87 | 13.06 | 20.13 | 4.59 | 0.17 | 0.87 | 0.19 |
| **Plt MaxOXPHOS** | 68.36 | 9.59 | 32.94 | 10.46 | 58.91 | 9.88 | 36.91 | 9.98 | 55.17 | 20.67 | 33.24 | 8.76 | 0.14 | 0.31 | 0.50 |
| **Plt MaxETS** | 114.94 | 19.40 | 56.30 | 18.71 | 120.21 | 19.99 | 65.15 | 19.35 | 96.39 | 29.71 | 55.15 | 15.89 | **0.05*** | 0.77 | 0.09 |

Supplemental Table 8: Differences in mitochondrial bioenergetic values between platelets obtained from normoglycemic (NG), prediabetic (PD) and diabetic (D) participants. PD and D comprised the IGT group. Significantly higher platelet mitochondrial bioenergetics is observed in the PD group compared to NG group in maximal respiration, spare respiratory capacities (SRC), and MaxETS respiration. Actual P values are reported. P values ≤ 0.05 are shown in bold with asterisk. * = ≤ 0.05

| **NG and IGT Separated** | **NG PLT (Unadjusted)** | | **IGT (Unadjusted)** | | **NG PLT (Age adjusted)** | | **IGT (Age adjusted)** | | **NG PLT (Sex adjusted)** | | **IGT (Sex adjusted)** | | **NG PLT (BMI adjusted)** | | **IGT (BMI adjusted)** | | **NG PLT (Age, Sex, BMI adjusted)** | | **IGT (Age, Sex, BMI adjusted)** | |
| --- | --- | --- | --- | --- | --- | --- | --- | --- | --- | --- | --- | --- | --- | --- | --- | --- | --- | --- | --- | --- |
|  | **OGTT_120 (R,P)** | **OGTT_AUC (R,P)** | **OGTT_120 (R,P)** | **OGTT_AUC (R,P)** | **OGTT_120 (R,P)** | **OGTT_AUC (R,P)** | **OGTT_120 (R,P)** | **OGTT_AUC (R,P)** | **OGTT_120 (R,P)** | **OGTT_AUC (R,P)** | **OGTT_120 (R,P)** | **OGTT_AUC (R,P)** | **OGTT_120 (R,P)** | **OGTT_AUC (R,P)** | **OGTT_120 (R,P)** | **OGTT_AUC (R,P)** | **OGTT_120 (R,P)** | **OGTT_AUC (R,P)** | **OGTT_120 (R,P)** | **OGTT_AUC (R,P)** |
| **Plt BASAL** | 0.11, 0.34 | 0.09, 0.40 | -0.21, 0.10 | -0.20, 0.14 | 0.08, 0.47 | 0.08, 0.49 | -0.18, 0.17 | -0.18, 0.18 | 0.07, 0.50 | 0.07, 0.55 | -0.12, 0.36 | -0.13, 0.34 | 0.10, 0.36 | 0.08, 0.47 | -0.21, 0.10 | -0.20, 0.14 | 0.02, 0.89 | 0.03, 0.83 | -0.10, 0.45 | -0.12, 0.39 |
| **Plt MAX** | 0.20, 0.10 | 0.11, 0.37 | -0.22, 0.10 | -0.20, 0.15 | 0.18, 0.15 | 0.10, 0.44 | -0.21, 0.12 | -0.20, 0.17 | 0.18, 0.13 | 0.09, 0.45 | -0.18, 0.18 | -0.17, 0.22 | 0.20, 0.10 | 0.11, 0.38 | -0.24, 0.08 | -0.22, 0.11 | 0.14, 0.27 | 0.06, 0.61 | -0.20, 0.15 | -0.20, 0.17 |
| **Plt SRC** | **0.22, 0.07** | 0.14, 0.25 | -0.14, 0.30 | -0.12, 0.39 | 0.19, 0.11 | 0.13, 0.30 | -0.15, 0.26 | -0.13, 0.37 | **0.21, 0.07** | 0.14, 0.26 | -0.16, 0.24 | -0.14, 0.33 | **0.22, 0.07** | 0.14, 0.25 | -0.16, 0.23 | -0.15, 0.28 | 0.18, 0.13 | 0.12, 0.35 | -0.20, 0.14 | -0.18, 0.22 |
| **Plt LEAK** | -0.19, 0.10 | -0.15, 0.20 | -0.03, 0.81 | -0.15, 0.29 | **-0.23*, 0.04** | -0.17, 0.14 | 0.00, 0.98 | -0.12, 0.37 | **-0.22*, 0.05** | -0.16, 0.16 | 0.02, 0.90 | -0.09, 0.50 | **-0.19, 0.09** | -0.16, 0.17 | -0.03, 0.80 | -0.15, 0.28 | **-0.27**, 0.01** | **-0.20, 0.09** | 0.04, 0.74 | -0.08, 0.58 |
| **Plt ATP-linked** | 0.15, 0.17 | 0.13, 0.25 | **-0.21, 0.09** | -0.19, 0.16 | 0.12, 0.29 | 0.11, 0.33 | -0.19, 0.14 | -0.17, 0.19 | 0.12, 0.27 | 0.10, 0.35 | -0.15, 0.25 | -0.14, 0.31 | 0.14, 0.19 | 0.12, 0.30 | **-0.21, 0.09** | -0.19, 0.16 | 0.06, 0.61 | 0.06, 0.60 | -0.13, 0.32 | -0.13, 0.36 |
| **Plt Coupling Efficiency** | **0.29**, 0.01** | **0.25*, 0.02** | 0.01, 0.96 | 0.15, 0.27 | **0.30**, 0.01** | **0.26*, 0.02** | -0.03, 0.82 | 0.13, 0.36 | **0.30**, 0.01** | **0.26*, 0.02** | -0.05, 0.73 | 0.09, 0.50 | **0.29**, 0.01** | **0.25*, 0.02** | 0.01, 0.95 | 0.15, 0.27 | **0.32**, 0.01** | **0.27*, 0.02** | -0.08, 0.56 | 0.07, 0.61 |
| **Plt FAO** | 0.05, 0.67 | 0.13, 0.24 | -0.04, 0.76 | -0.18, 0.21 | 0.02, 0.89 | 0.13, 0.27 | -0.03, 0.84 | -0.17, 0.23 | 0.05, 0.66 | 0.13, 0.24 | -0.04, 0.76 | -0.18, 0.20 | 0.05, 0.68 | 0.13, 0.24 | -0.05, 0.73 | -0.18, 0.19 | 0.02, 0.89 | 0.13, 0.27 | -0.04, 0.75 | -0.19, 0.19 |
| **Plt FAO+CI** | 0.15, 0.16 | **0.22*, 0.04** | -0.08, 0.57 | -0.22, 0.12 | 0.12, 0.26 | **0.22*, 0.05** | -0.08, 0.54 | -0.21, 0.13 | 0.16, 0.14 | **0.22*, 0.04** | -0.07, 0.61 | -0.20, 0.16 | 0.15, 0.16 | **0.23*, 0.04** | -0.08, 0.56 | -0.21, 0.13 | 0.14, 0.22 | **0.22*, 0.04** | -0.09, 0.53 | -0.22, 0.12 |
| **Plt FAO+CI+CII** | **0.24*, 0.02** | **0.25*, 0.02** | -0.07, 0.63 | -0.12, 0.37 | **0.21*, 0.05** | **0.23*, 0.03** | -0.08, 0.56 | -0.14, 0.33 | **0.25*, 0.02** | **0.24*, 0.03** | -0.06, 0.65 | -0.12, 0.39 | **0.24*, 0.02** | **0.24*, 0.03** | -0.07, 0.63 | -0.13, 0.37 | **0.22*, 0.04** | **0.23*, 0.04** | -0.08, 0.56 | -0.14, 0.33 |
| **Plt MaxOXPHOS** | **0.19, 0.08** | 0.14, 0.20 | -0.11, 0.41 | -0.17, 0.22 | 0.15, 0.18 | 0.13, 0.24 | -0.11, 0.40 | -0.17, 0.22 | **0.20, 0.06** | 0.14, 0.21 | -0.11, 0.42 | -0.17, 0.23 | **0.19, 0.08** | 0.14, 0.21 | -0.11, 0.41 | -0.17, 0.22 | 0.16, 0.14 | 0.13, 0.24 | -0.12, 0.40 | -0.17, 0.22 |
| **Plt MaxETS** | **0.19, 0.08** | **0.19, 0.08** | -0.16, 0.22 | -0.21, 0.13 | 0.15, 0.18 | **0.19, 0.09** | -0.16, 0.24 | -0.21, 0.14 | **0.20, 0.06** | **0.19, 0.09** | -0.17, 0.23 | -0.21, 0.13 | **0.19, 0.08** | **0.20, 0.07** | -0.17, 0.22 | -0.21, 0.13 | 0.16, 0.15 | **0.19, 0.09** | -0.17, 0.23 | -0.21, 0.14 |

Supplemental Table 9: Spearman correlation coefficients (R) and P values assessed between platelet mitochondrial bioenergetic parameters and OGTT_120 and OGTT_AUC values in NG and IGT participants grouped separately are shown in the first 2 columns (unadjusted). Columns 3, 4, 5, 6, 7, 8, 9, and 10 show partial correlation coefficients (R) and P values assessed in NG and IGT participants grouped separately between platelet mitochondrial bioenergetic parameters and OGTT_120 and OGTT_AUC values after individual adjustments with age, sex, and BMI, and after adjusting for age, sex, and BMI together.

| **PD and D Separated** | **PD PLT (Unadjusted)** | | **D (Unadjusted)** | | **PD PLT (Age adjusted)** | | **D (Age adjusted)** | | **PD PLT (Sex adjusted)** | | **D (Sex adjusted)** | | **PD PLT (BMI adjusted)** | | **D (BMI adjusted)** | | **PD PLT (Age, Sex, BMI adjusted)** | | **D (Age, Sex, BMI adjusted)** | |
| --- | --- | --- | --- | --- | --- | --- | --- | --- | --- | --- | --- | --- | --- | --- | --- | --- | --- | --- | --- | --- |
|  | **OGTT_120 (R,P)** | **OGTT_AUC (R,P)** | **OGTT_120 (R,P)** | **OGTT_AUC (R,P)** | **OGTT_120 (R,P)** | **OGTT_AUC (R,P)** | **OGTT_120 (R,P)** | **OGTT_AUC (R,P)** | **OGTT_120 (R,P)** | **OGTT_AUC (R,P)** | **OGTT_120 (R,P)** | **OGTT_AUC (R,P)** | **OGTT_120 (R,P)** | **OGTT_AUC (R,P)** | **OGTT_120 (R,P)** | **OGTT_AUC (R,P)** | **OGTT_120 (R,P)** | **OGTT_AUC (R,P)** | **OGTT_120 (R,P)** | **OGTT_AUC (R,P)** |
| **Plt BASAL** | -0.18, 0.22 | -0.23, 0.12 | -0.43, 0.13 | -0.08, 0.80 | -0.13, 0.38 | -0.21, 0.16 | -0.48, 0.10 | 0.04, 0.90 | -0.16, 0.26 | -0.20, 0.18 | -0.20, 0.52 | 0.03, 0.93 | -0.15, 0.31 | -0.23, 0.13 | -0.37, 0.22 | -0.01, 0.98 | -0.10, 0.49 | -0.19, 0.23 | -0.26, 0.45 | 0.19, 0.63 |
| **Plt MAX** | -0.23, 0.13 | -0.19, 0.23 | -0.33, 0.26 | -0.18, 0.58 | -0.21, 0.19 | -0.18, 0.26 | -0.36, 0.22 | -0.08, 0.81 | -0.23, 0.15 | -0.19, 0.24 | -0.10, 0.73 | -0.10, 0.77 | -0.20, 0.20 | -0.23, 0.15 | -0.28, 0.36 | -0.13, 0.71 | -0.18, 0.26 | -0.22, 0.18 | -0.18, 0.61 | 0.01, 0.97 |
| **Plt SRC** | -0.15, 0.33 | -0.06, 0.70 | -0.07, 0.81 | -0.25, 0.44 | -0.17, 0.28 | -0.07, 0.68 | -0.08, 0.79 | -0.21, 0.53 | -0.16, 0.30 | -0.09, 0.60 | 0.02, 0.94 | -0.21, 0.53 | -0.12, 0.44 | -0.10, 0.56 | -0.09, 0.78 | -0.23, 0.49 | -0.15, 0.34 | -0.13, 0.44 | -0.03, 0.92 | -0.17, 0.66 |
| **Plt LEAK** | -0.05, 0.75 | -0.25, 0.10 | -0.13, 0.68 | -0.11, 0.77 | 0.01, 0.97 | -0.23, 0.14 | -0.18, 0.59 | -0.05, 0.91 | -0.03, 0.84 | -0.21, 0.16 | -0.01, 0.98 | 0.05, 0.91 | -0.01, 0.96 | -0.25, 0.10 | -0.05, 0.89 | -0.03, 0.94 | 0.04, 0.77 | -0.20, 0.20 | -0.004, 0.99 | 0.02, 0.97 |
| **Plt ATP-linked** | -0.19, 0.20 | -0.20, 0.19 | **-0.48, 0.08** | -0.12, 0.71 | -0.14, 0.33 | -0.18, 0.24 | **-0.54*, 0.05** | 0.03, 0.94 | -0.18, 0.23 | -0.18, 0.24 | -0.27, 0.38 | -0.02, 0.96 | -0.17, 0.26 | -0.20, 0.19 | -0.43, 0.15 | -0.06, 0.86 | -0.13, 0.40 | -0.16, 0.29 | -0.36, 0.27 | 0.19, 0.63 |
| **Plt Coupling Efficiency** | -0.01, 0.95 | 0.21, 0.16 | 0.11, 0.73 | 0.19, 0.61 | -0.06, 0.71 | 0.19, 0.20 | 0.14, 0.69 | 0.16, 0.68 | -0.03, 0.84 | 0.17, 0.25 | -0.03, 0.94 | 0.04, 0.93 | -0.04, 0.80 | 0.21, 0.16 | -0.01, 0.97 | 0.10, 0.80 | -0.09, 0.57 | 0.16, 0.31 | -0.13, 0.74 | 0.18, 0.69 |
| **Plt FAO** | -0.14, 0.35 | -0.22, 0.16 | 0.26, 0.42 | -0.13, 0.70 | -0.14, 0.36 | -0.22, 0.17 | 0.36, 0.27 | 0.08, 0.84 | -0.14, 0.36 | -0.22, 0.16 | 0.28, 0.41 | -0.13, 0.72 | -0.09, 0.55 | -0.23, 0.15 | 0.11, 0.75 | -0.10, 0.79 | -0.09, 0.56 | -0.23, 0.15 | 0.37, 0.33 | 0.14, 0.73 |
| **Plt FAO+CI** | -0.18, 0.24 | -0.22, 0.17 | 0.32, 0.31 | -0.19, 0.59 | -0.19, 0.21 | -0.22, 0.17 | 0.38, 0.25 | -0.07, 0.85 | -0.18, 0.24 | -0.21, 0.19 | 0.31, 0.36 | -0.19, 0.59 | -0.18, 0.25 | -0.22, 0.16 | 0.24, 0.48 | -0.17, 0.65 | -0.20, 0.21 | -0.22, 0.17 | 0.36, 0.35 | -0.08, 0.85 |
| **Plt FAO+CI+CII** | -0.12, 0.43 | -0.09, 0.57 | 0.10, 0.76 | -0.32, 0.33 | -0.14, 0.37 | -0.10, 0.55 | 0.12, 0.72 | -0.29, 0.41 | -0.12, 0.43 | -0.09, 0.58 | 0.15, 0.65 | -0.31, 0.38 | -0.12, 0.45 | -0.10, 0.55 | 0.10, 0.76 | -0.33, 0.35 | -0.13, 0.40 | -0.11, 0.53 | 0.28, 0.47 | -0.21, 0.62 |
| **Plt MaxOXPHOS** | -0.16, 0.28 | -0.15, 0.32 | -0.13, 0.69 | **-0.53, 0.09** | -0.17, 0.27 | -0.16, 0.33 | -0.10, 0.79 | -0.49, 0.16 | -0.16, 0.29 | -0.15, 0.33 | -0.10, 0.86 | -0.52, 0.12 | -0.14, 0.37 | -0.16, 0.32 | -0.12, 0.73 | -0.54, 0.11 | -0.14, 0.36 | -0.16, 0.33 | 0.15, 0.70 | -0.38, 0.35 |
| **Plt MaxETS** | -0.20, 0.19 | -0.16, 0.30 | -0.10, 0.79 | -0.49, 0.12 | -0.20, 0.19 | -0.16, 0.30 | -0.10, 0.88 | -0.46, 0.19 | -0.20, 0.20 | -0.17, 0.30 | -0.02, 0.96 | -0.48, 0.16 | -0.16, 0.29 | -0.17, 0.28 | -0.07, 0.84 | -0.51, 0.14 | -0.16, 0.30 | -0.17, 0.29 | 0.17, 0.66 | -0.36, 0.39 |

Supplemental Table 10: Spearman correlation coefficients (R) and P values assessed between platelet mitochondrial bioenergetic parameters and OGTT_120 and OGTT_AUC values in prediabetic (PD) and diabetic (D) participants grouped separately are shown in the first 2 columns (unadjusted). Columns 3, 4, 5, 6, 7, 8, 9, and 10 show partial correlation coefficients (R) and P values assessed in PD and D participants grouped separately between platelet mitochondrial bioenergetic parameters and OGTT_120 and OGTT_AUC values after individual adjustments with age, sex, and BMI, and after adjusting for age, sex, and BMI together.

| **PD and D Separated** |  | | | | | | | | | |
| --- | --- | --- | --- | --- | --- | --- | --- | --- | --- | --- |
|  | **Unadjusted** | | **Age adjusted** | | **Sex adjusted** | | **BMI adjusted** | | **Age, Sex, BMI adjusted** | |
|  | **PD (R,P)** | **D (R,P)** | **PD (R,P)** | **D (R,P)** | **PD (R,P)** | **D (R,P)** | **PD (R,P)** | **D (R,P)** | **PD (R,P)** | **D (R,P)** |
| **Plt BASAL** | **0.40*, 0.02** | -0.02, 0.92 | 0.23, 0.22 | 0.02, 0.94 | **0.35*, 0.05** | -0.15, 0.52 | **0.36*, 0.04** | 0.06, 0.79 | 0.10, 0.61 | -0.004, 0.99 |
| **Plt MAX** | 0.25, 0.17 | 0.01, 0.97 | 0.14, 0.47 | 0.09, 0.72 | 0.23, 0.22 | -0.11, 0.67 | 0.18, 0.34 | 0.04, 0.90 | 0.01, 0.98 | -0.04, 0.89 |
| **Plt SRC** | 0.06, 0.76 | 0.09, 0.73 | 0.05, 0.80 | 0.10, 0.69 | 0.08, 0.69 | 0.06, 0.82 | -0.01, 0.97 | -0.02, 0.95 | -0.01, 0.98 | -0.04, 0.90 |
| **Plt LEAK** | 0.22, 0.24 | **-0.40, 0.08** | 0.05, 0.80 | **-0.38, 0.10** | 0.17, 0.38 | **-0.44*, 0.05** | 0.15, 0.44 | -0.25, 0.30 | -0.12, 0.54 | -0.30, 0.24 |
| **Plt ATP-linked** | **0.38*, 0.03** | 0.02, 0.95 | 0.24, 0.20 | 0.06, 0.79 | **0.34, 0.06** | -0.10, 0.68 | **0.35*, 0.05** | 0.07, 0.76 | 0.14, 0.47 | 0.03, 0.91 |
| **Plt Coupling Efficiency** | -0.12, 0.50 | 0.38, 0.09 | 0.02, 0.92 | 0.37, 0.11 | -0.07, 0.72 | 0.38, 0.10 | -0.07, 0.71 | 0.24, 0.32 | 0.16, 0.42 | 0.29, 0.26 |
| **Plt FAO** | 0.14, 0.48 | 0.02, 0.92 | 0.13, 0.50 | 0.03, 0.88 | 0.16, 0.41 | 0.06, 0.78 | 0.08, 0.69 | -0.02, 0.94 | 0.10, 0.62 | 0.04, 0.89 |
| **Plt FAO+CI** | -0.06, 0.77 | -0.01, 0.95 | 0.03, 0.90 | 0.01, 0.96 | -0.07, 0.73 | 0.003, 0.99 | -0.07, 0.71 | -0.03, 0.89 | -0.00, 0.97 | -0.01, 0.96 |
| **Plt FAO+CI+CII** | -0.03, 0.88 | -0.14, 0.53 | 0.06, 0.76 | -0.13, 0.58 | -0.02, 0.93 | -0.14, 0.54 | -0.01, 0.94 | -0.11, 0.64 | 0.07, 0.74 | -0.11, 0.67 |
| **Plt MaxOXPHOS** | -0.07, 0.71 | -0.14, 0.51 | -0.02, 0.90 | -0.14, 0.53 | -0.07, 0.81 | -0.15, 0.50 | -0.09, 0.66 | -0.15, 0.52 | -0.03, 0.89 | -0.16, 0.52 |
| **Plt MaxETS** | 0.05, 0.80 | -0.14, 0.52 | 0.06, 0.76 | -0.12, 0.59 | 0.07, 0.72 | -0.15, 0.50 | 0.03, 0.90 | -0.11, 0.63 | 0.05, 0.81 | -0.13, 0.60 |

Supplemental Table 11: Spearman correlation coefficients (R) and P values assessed between platelet mitochondrial bioenergetic parameters and HbA1c values in prediabetic (PD) and diabetic (D) participants grouped separately are shown in the first 2 columns (unadjusted). Columns 2, 3, 4, and 5 show partial correlation coefficients (R) and P values assessed in PD and D participants grouped separately between platelet mitochondrial bioenergetic parameters and HbA1c values after individual adjustments with age, sex, and BMI, and after adjusting for age, sex, and BMI together.
