# Supplementary figures and images for: Platelets from older adults exhibit differences in mitochondrial function associated with impaired glucose metabolism
Source: Clin Sci (Lond). 2026 Jan 14;140(1):65–79. doi: 10.1042/CS20242841 (PMC12862959; doi:10.1042/CS20242841)

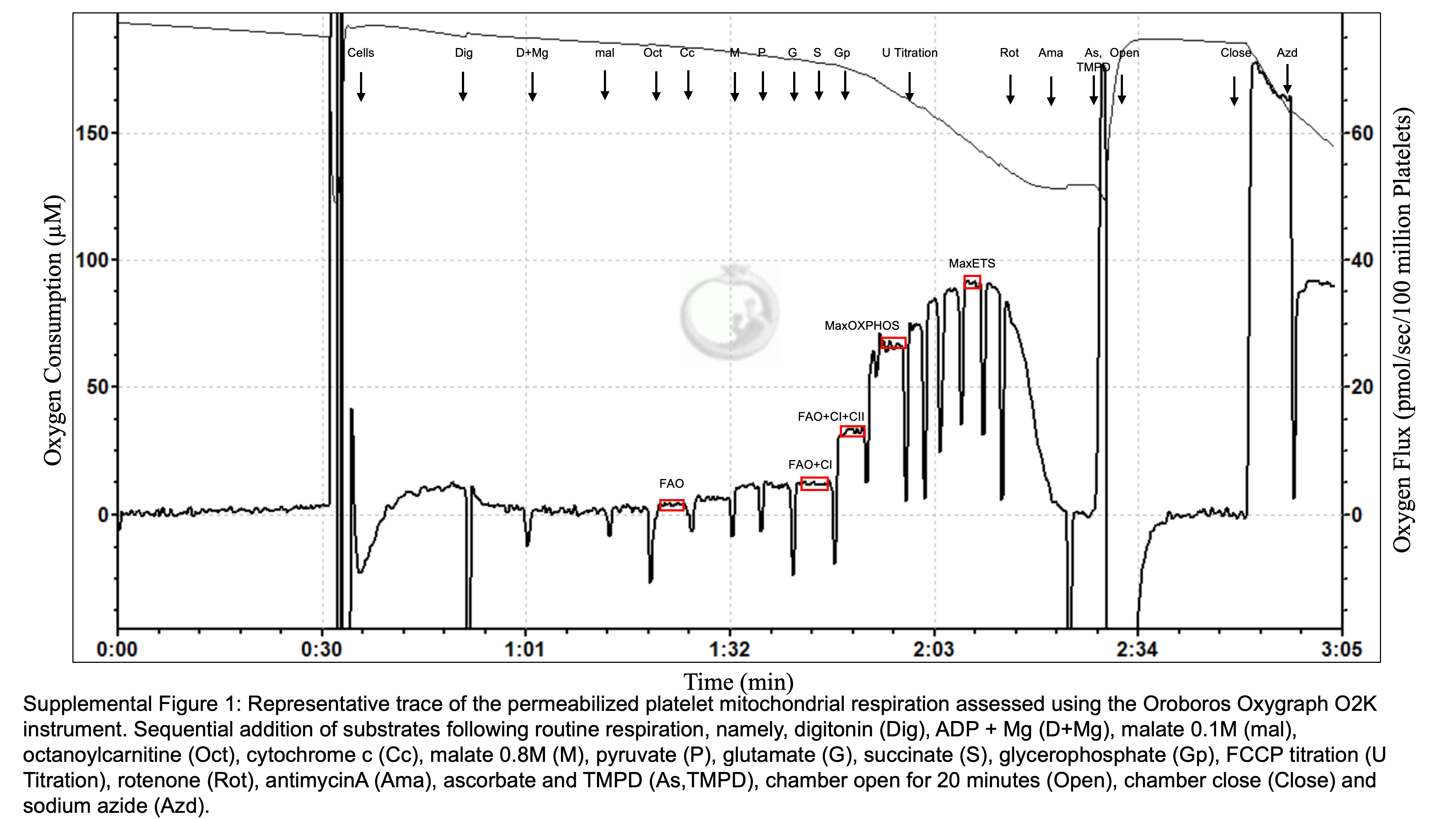

Supplement: online supplementary figure 1. [file cs-140-1-CS20242841-s001.tif]

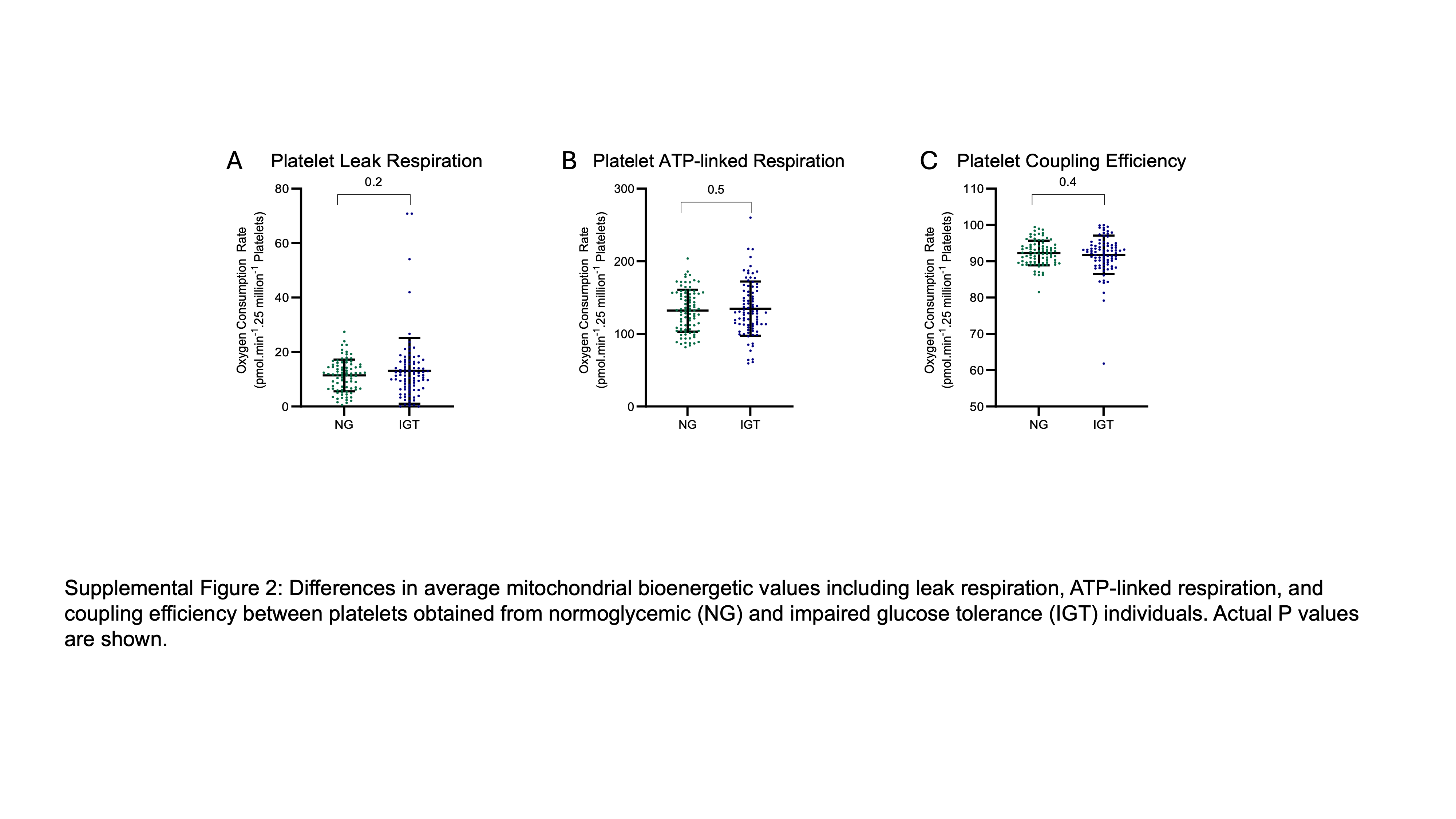

Supplement: online supplementary figure 2. [file cs-140-1-CS20242841-s002.tif]
